# Supplementary material for: Targeting fatty acid oxidation enhances response to HER2-targeted therapy
Source: Nat Commun. 2024 Aug 3;15:6587. doi: 10.1038/s41467-024-50998-3 (PMC11297952; doi:10.1038/s41467-024-50998-3)
Supplement: Supplementary file 1 — Supplementary Information [file 41467_2024_50998_MOESM1_ESM.docx]

**Supplementary Information**

**Targeting Fatty Acid Oxidation Enhances Response to HER2-targeted Therapy**

Nandi et al.

Supplementary Figures 1-12

Supplementary Tables 1-4

**Supplementary Figures**

**
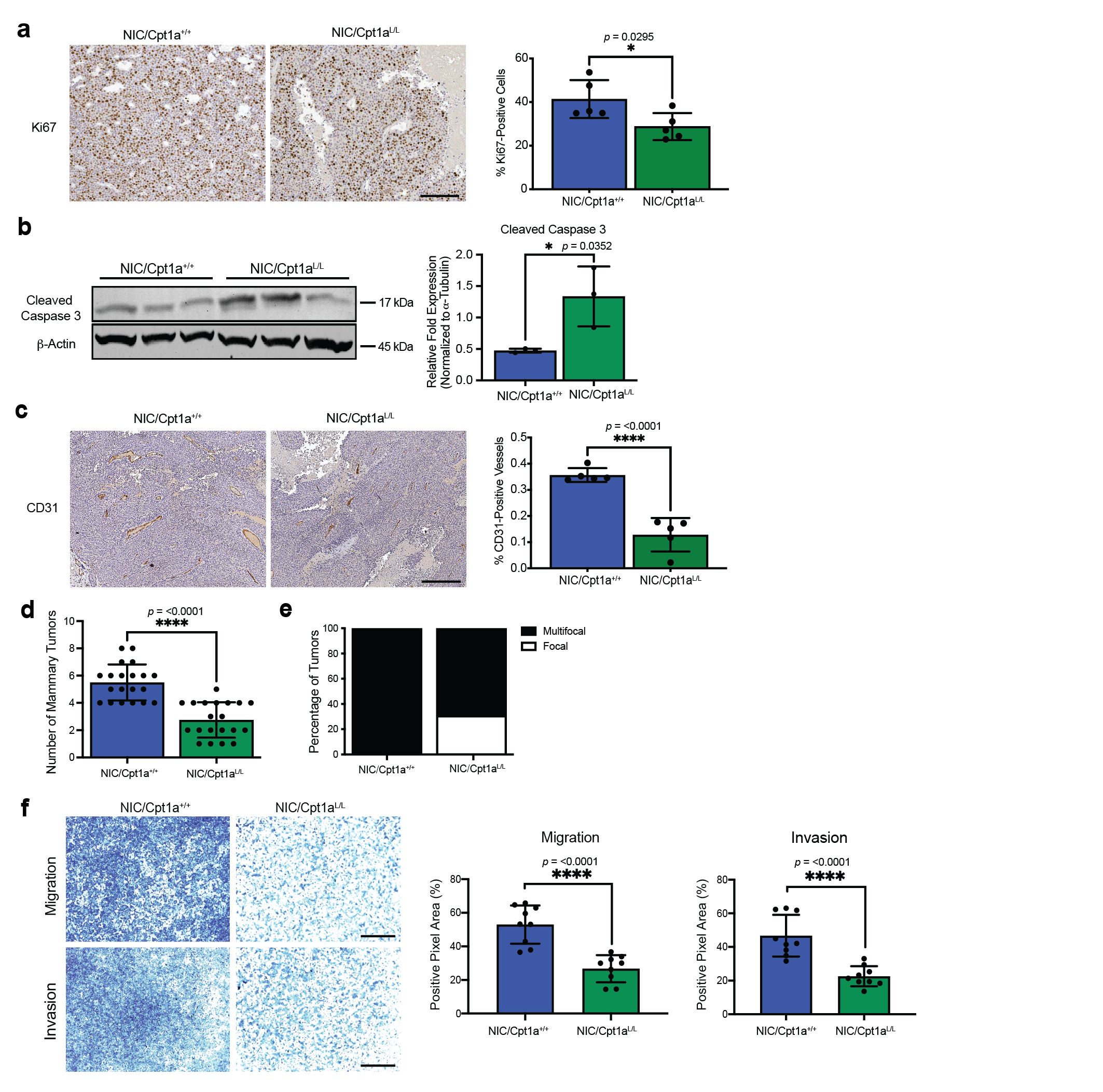
Supplementary Figure 1: Cpt1a-deficient NIC Tumors are Necrotic and Exhibit Decreased Blood Vessel Diameter**

**a,** Control and Cpt1a-deficient tumors were stained for Ki67, a proliferation marker, expression by immunohistochemistry (IHC). Left panel - Representative images. Scale bar represents 100 µm. Right panel - Quantification of the percentage of Ki67-positive nuclei in tumors. *n* = 5 mice per genotype (minimum 10,000 total nuclei analyzed per mouse). **p* < 0.05; unpaired, two-tailed Student’s t-test. **b,** Lysates of NIC/Cpt1a^+/+^ and NIC/Cpt1a^L/L^  cells were immunoblotted with the indicated antibodies. Representative immunoblots and quantification (fluorescent immunoblotting – LiCOR Odyssey) of Cleaved Caspase 3 expression are shown. 3 independent cell lines were analyzed per genotype. **p* < 0.05; unpaired, two-tailed Student’s t-test. **c,** Tumors as in (a) were stained for stained for CD31, a marker of endothelial cells, by IHC. Left panel - Representative images. Scale bar represents 100 µm. Right panel - Quantification of the percentage of CD31-positive vessels in tumors. *n* = 5 mice per genotype (minimum 10,000 total nuclei analyzed per mouse). *****p* < 0.0001; unpaired, two-tailed Student’s t-test. **d-e,** Quantification of the total number of mammary tumors per mouse (d) and percentage of focal and multifocal tumors, for each genotype. *n* = 20 mice per genotype - *****p* < 0.0001; unpaired, two-tailed Student’s t-test. **f,** Left panel –Representative images of cell migration and invasion (Boyden chamber) assays. Scale bar represents 1000 μm. Right Panel – quantification (positive pixel area) of cell migration and invasion (n = 3 cell lines per genotype in triplicate – *****p* < 0.0001; unpaired, two-tailed Student’s *t*-test). All error bars are expressed as mean values ± SD. Source data are provided as a Source Data file.

**
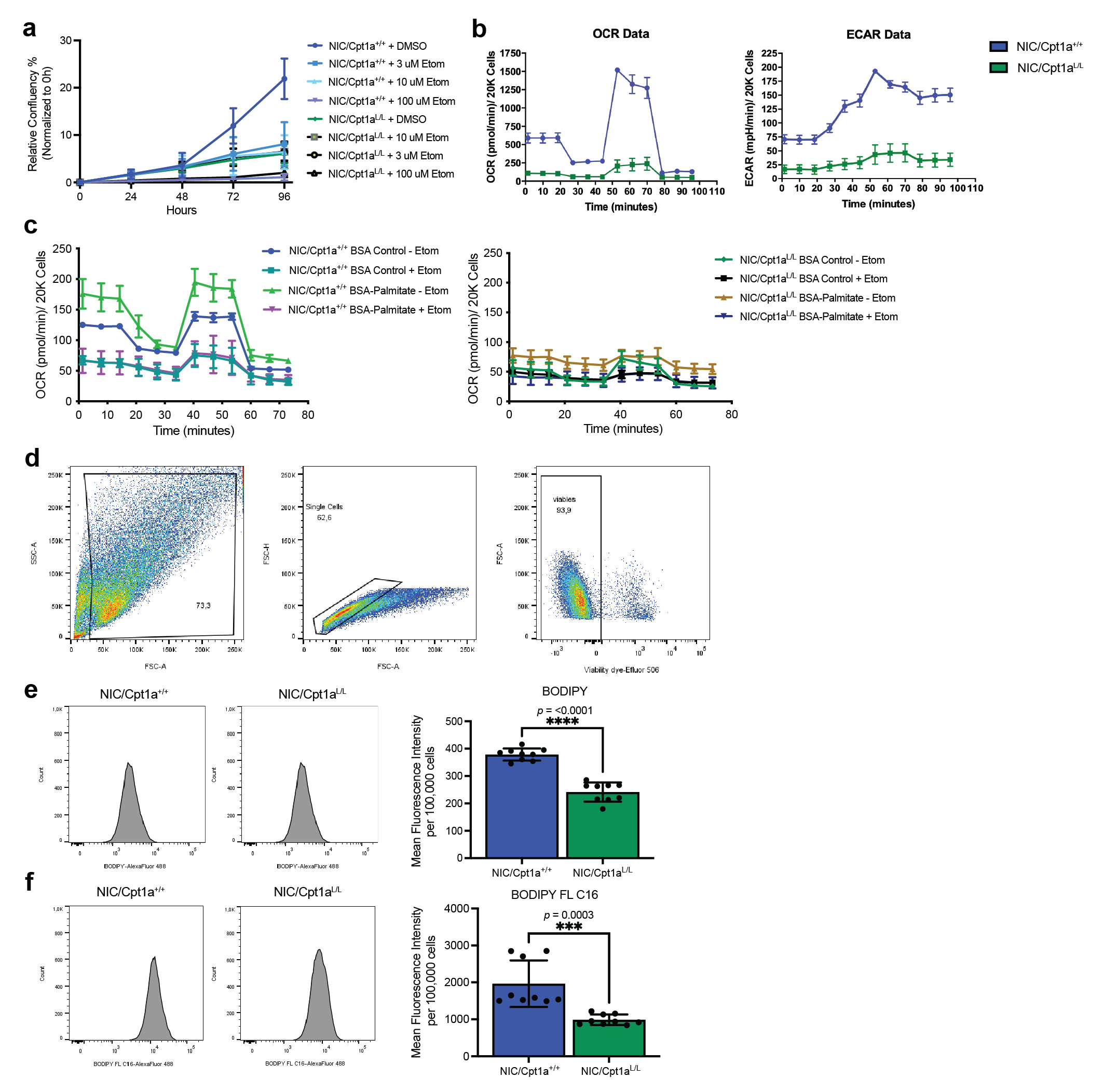
Supplementary Figure 2: Deletion of Cpt1a impairs OXPHOS, glycolysis and utilization of exogenous fatty acids for FAO.**

**a,** Growth curves from the proliferation assay Figure 2b. **b,** Left panel - Basal, maximal (FCCP), ATP-synthesis coupled (Oligomycin A), and non-mitochondrial (rotenone/ antimycin A) oxygen consumption rates (OCRs) of NIC/Cpt1a^+/+^ and NIC/Cpt1a^L/L^ cells. Representative of *n* = 3 cell lines. Right panel - pH changes per minute in the cell culture medium measured simultaneously with OCR, Representative of *n* = 3 cell lines per genotype. **c,** Left panel - Basal, maximal (FCCP), ATP-synthesis coupled (Oligomycin A), and non-mitochondrial (rotenone/ antimycin A) OCRs of NIC/Cpt1a^+/+^ and NIC/ Cpt1a^L/L^ cells in the presence of bovine serum albumin (BSA)-control or palmitate and in the presence or absence of Etomoxir (Etom). Representative of *n* = 3 cell lines. Right panel - pH changes per minute in the cell culture medium from cells as 2f measured simultaneously with OCR, Representative of *n* = 3 cell lines per genotype. **d,** Representative gating strategy for flow cytometry analysis of (e-f) assessing BODIPY 493/503 and BODIPY FL C16 (Alexa Fluor 488) in viable (eFluor 506-positive) NIC/Cpt1a^+/+^ and NIC/Cpt1a^L/L^ cells. **e-f,** Representative histograms (Left panel) and mean fluorescence intensity (MFI) (Right panel) of BODIPY 493/503 (d) and BODIPY FL C16 (e) incorporation in NIC/ Cpt1a^+/+^ and NIC/ Cpt1a^L/L^ cells. *n* = 3 cell lines per genotype analyzed in triplicate -****p*< 0.001, *****p* < 0.0001; unpaired, two-tailed Student’s t-test. All error bars are expressed as mean values ± SD. Source data are provided as a Source Data file.

**
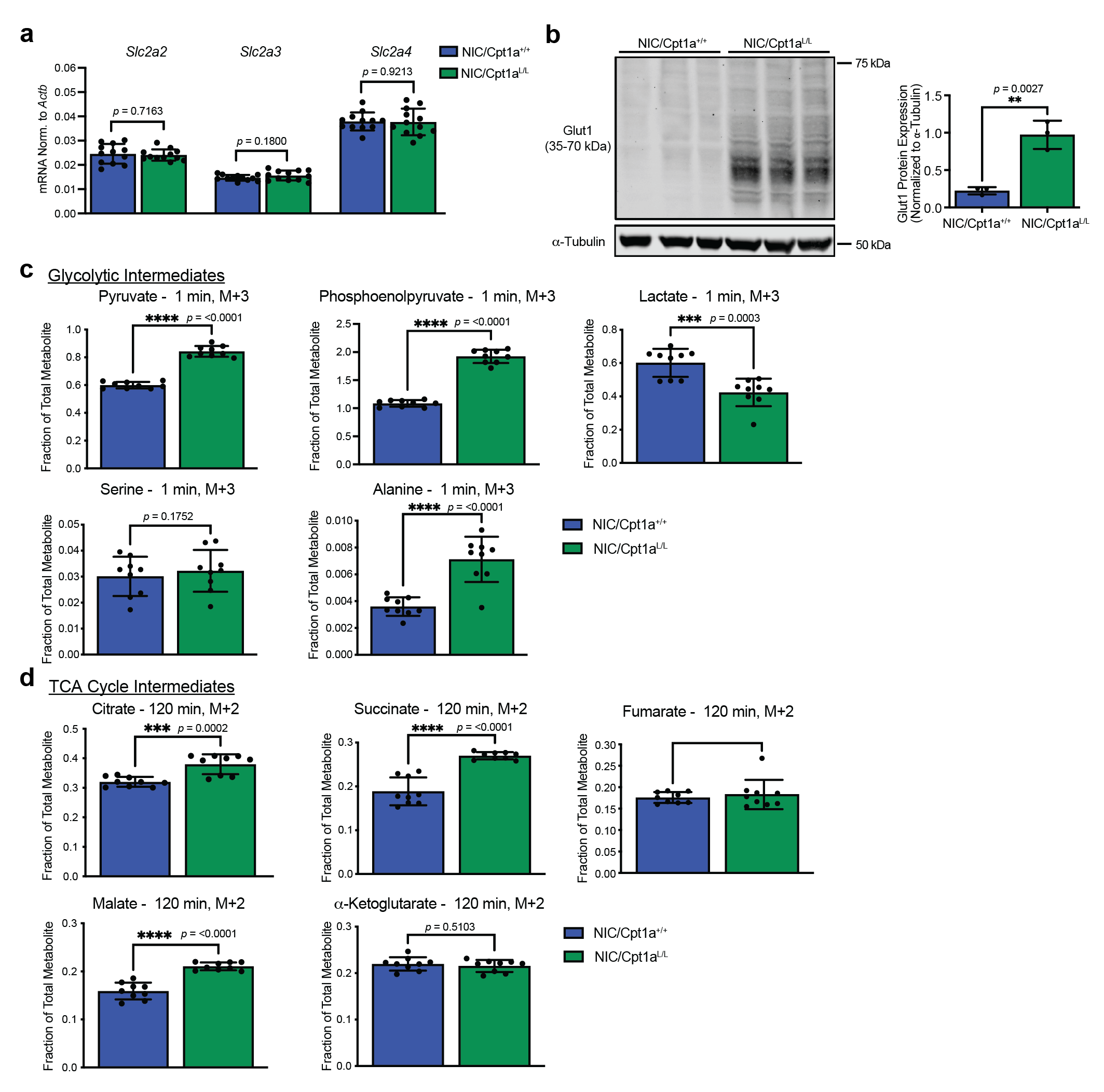
Supplementary Figure 3: U-^13^C-glucose labeling shows loss of Cpt1a drives synthesis of TCA cycle intermediates from glucose**

**a,** QRT-PCR analysis of Glut2 (*Slc2a2*), Glut3 (*Slc2a3*) and Glut4 (*Slc2a4*) in NIC/Cpt1a^+/+^ and NIC/Cpt1a^L/L^ cells. Expression was normalized to that of *Actb*. *n* = 4 cell lines per genotype in triplicate - ****p*< 0.001, *****p* < 0.0001; unpaired, two-tailed Student’s t-test. **b,** NIC/Cpt1a^+/+^ and NIC/Cpt1a^L/L^  lysates were immunoblotted with the indicated antibodies. Representative immunoblots and quantification (fluorescent immunoblotting – LiCOR Odyssey) of Glut1 expression are shown. 3 independent cell lines were analyzed per genotype in triplicate. ***p* < 0.01; unpaired, two-tailed Student’s t-test. **c-d,** Fractional ion abundance of Glycolytic (Pyruvate, Phosphoenolpyruvate, Lactate, Serine and Alanine) and TCA Cycle (Citrate, Succinate, Fumarate, Malate and a-KG) intermediates following a pulse with U-^13^C-glucose at the indicated time points of NIC/Cpt1a^+/+^ and NIC/Cpt1a^L/L^  cells. *n* = 3 per genotype, analyzed in triplicate - ****p* < 0.001, *****p* < 0.0001; unpaired, two-tailed Student’s t-test. All error bars are expressed as mean values ± SD. Source data are provided as a Source Data file.

**
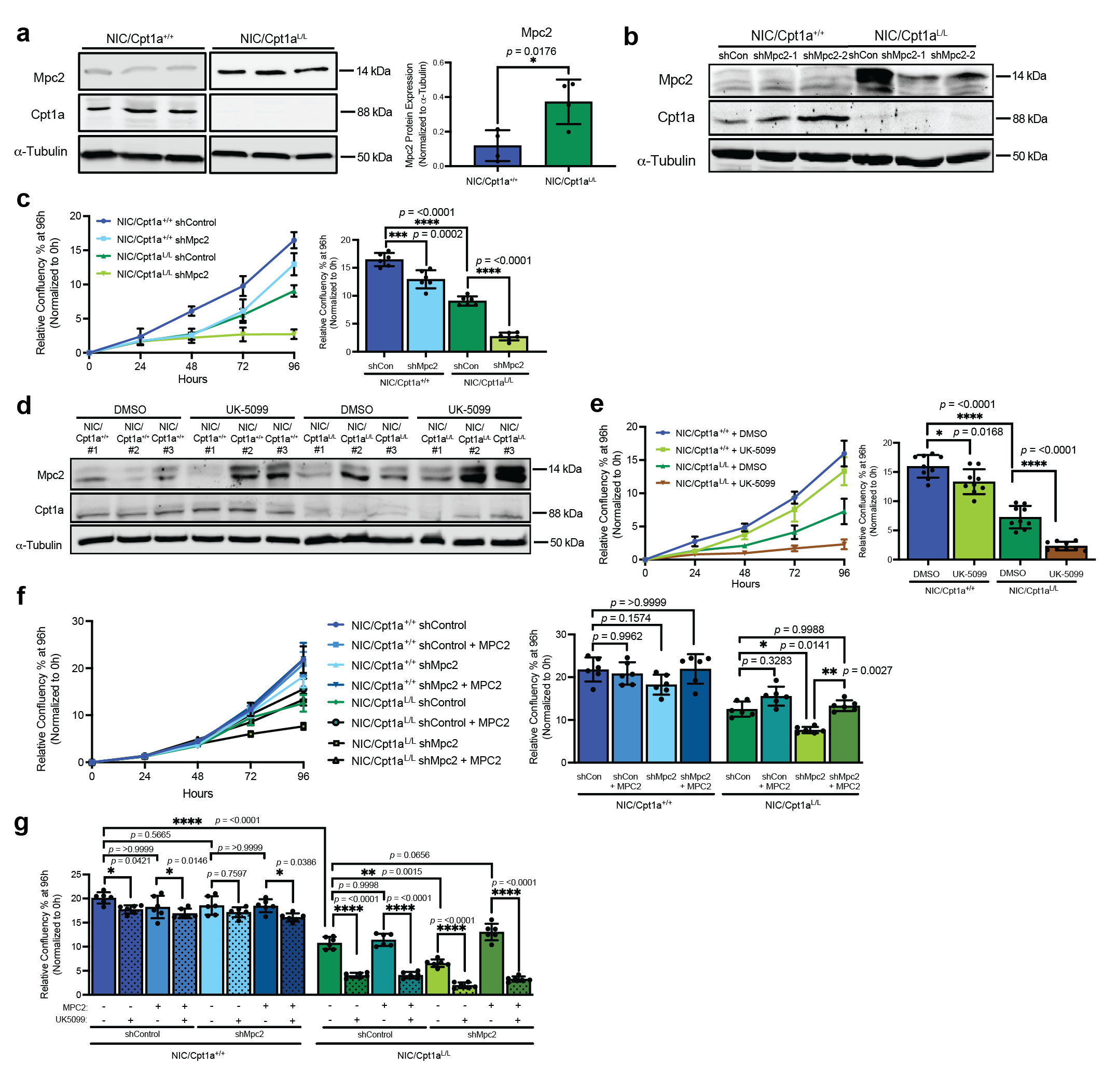
Supplementary Figure 4: Ablation of Cpt1a confers dependence on pyruvate transport into the mitochondria.**

**a,** NIC/Cpt1a^+/+^ and NIC/Cpt1a^L/L^ lysates were immunoblotted with the indicated antibodies. Representative immunoblots (Left panel) and quantification (fluorescent immunoblotting – LiCOR Odyssey) (Right panel) of Mpc2 expression are shown. 4 independent cell lines were analyzed per genotype. **p* < 0.05; unpaired, two-tailed Student’s t-test. **b,** NIC/Cpt1a^+/+^ and NIC/Cpt1a^L/L^ cells stably expressing short hairpin RNAs (shRNAs) against luciferase (control - shCon) or Mpc2 (*Brp44*) were transduced *in vitro* and immunoblotted with the indicated antibodies.  **c,** Proliferation was assessed from cells as in (b) using an imaging-based assay to monitor cell confluency in real time. Left panel – growth curves. Right panel – endpoint analysis of cell growth at 96h. Data were normalized to confluency at t=0. *n* = 2 cell lines in triplicate - ****p* < 0.001, *****p* < 0.0001; unpaired, two-tailed Student’s t-test**. d,** Lysates of NIC/Cpt1a^+/+^ and NIC/Cpt1a^L/L^  cells were immunoblotted with the indicated antibodies. Mitochondrial pyruvate carrier (MPC) inhibitor, UK5099, was used at 10μM. **e,** NIC cells were treated with 10μM UK5099, or DMSO as a control. Proliferation was assessed by Incucyte® to measure cell confluency in real time. Growth curves (left panel) correspond to endpoint data at 96 hours (right panel). *n* = 3 per genotype, analyzed in triplicate - **p* < 0.05 and *****p* < 0.0001; one-way ANOVA with Tukey’s post-hoc test. **f,** Left panel - Proliferation assay of NIC/Cpt1a^+/+^ and NIC/Cpt1a^L/L^ shControl and shMpc2 cells stably transduced with human MPC2 constructs, resistant to targeting my shMpc2, assessed by Incucyte®. Right panel – Percentage relative confluency of cells at 96 hours, normalized to t=0. (*n=*3 cell lines) (**p* < 0.05, ***p* < 0.05; one-way ANOVA with Tukey’s post-hoc test). **g,** Growth curve (left panel) and end-point proliferation data (right panel) of NIC shControl and shMpc2 cells stably transduced with human MPC2 and treated with UK5099 (10μM) at 96 hours, normalized to t=0. (*n=*3 cell lines) (***p* < 0.01, *****p* < 0.0001; one-way ANOVA with Tukey’s post-hoc test). All error bars are expressed as mean values ± SD. Source data are provided as a Source Data file.

**
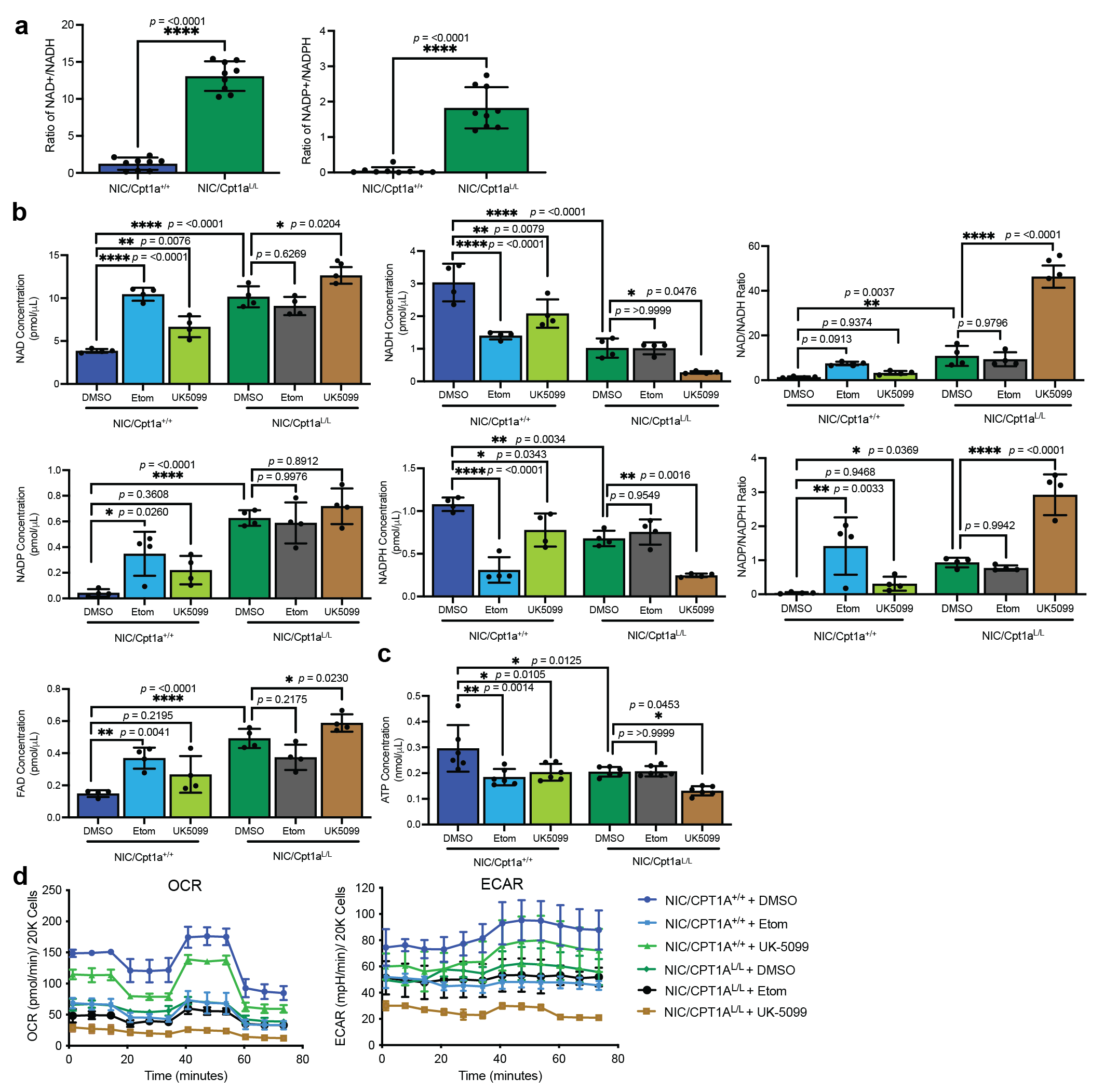
Supplementary Figure 5: Acetyl CoA from FAs drives TCA flux in wild-type ErbB2+ breast cancer cells while Cpt1a-deficient cells are dependent on pyruvate.**

**a,** Ratio of NAD/NADH (left) and NADP/NADPH (right) in NIC/Cpt1a^+/+^ and NIC/Cpt1a^L/L^ cells. *n* = 4 per genotype, analyzed in triplicate - **p* < 0.05, ***p* < 0.01; unpaired, two-tailed Student’s t-test. **b,** Relative abundance of oxidized (nicotinamide adenine dinucleotide - NAD^+^, nicotinamide adenine dinucleotide phosphate - NADP^+^, and flavin adenine dinucleotide - FAD) and reduced (NADH and NADPH) forms of electron carriers in NIC/Cpt1a^+/+^ and NIC/Cpt1a^L/L^  cells treated with 3μM Etomoxir (Etom), 10 μM MPC inhibitor, UK-5099, or DMSO as a control. *n* = 2 per genotype, analyzed in duplicate - **p* < 0.05, ***p* < 0.01, ****p* < 0.001, *****p* < 0.0001; one-way ANOVA with Tukey’s post-hoc test. **c,** Relative ATP concentration in NIC/Cpt1a^+/+^ and NIC/Cpt1a^L/L^ cells treated with DMSO, Etomoxir and UK-5099. *n* = 3 per genotype, analyzed in triplicate -**p* < 0.05, ***p* < 0.001; one-way ANOVA with Tukey’s post-hoc test. **d,** Left panel - Basal, maximal (FCCP), ATP-synthesis coupled (Oligomycin A), and non-mitochondrial (rotenone/ antimycin A) oxygen consumption rates (OCRs) of NIC/Cpt1a^+/+^ and NIC/Cpt1a^L/L^ cells treated with DMSO, Etomoxir and UK-5099. Representative of *n* = 3 cell lines. Right panel - pH changes per minute in the cell culture medium measured simultaneously with OCR, Representative of *n* = 3 cell lines per genotype. All error bars are expressed as mean values ± SD. Source data are provided as a Source Data file.

**
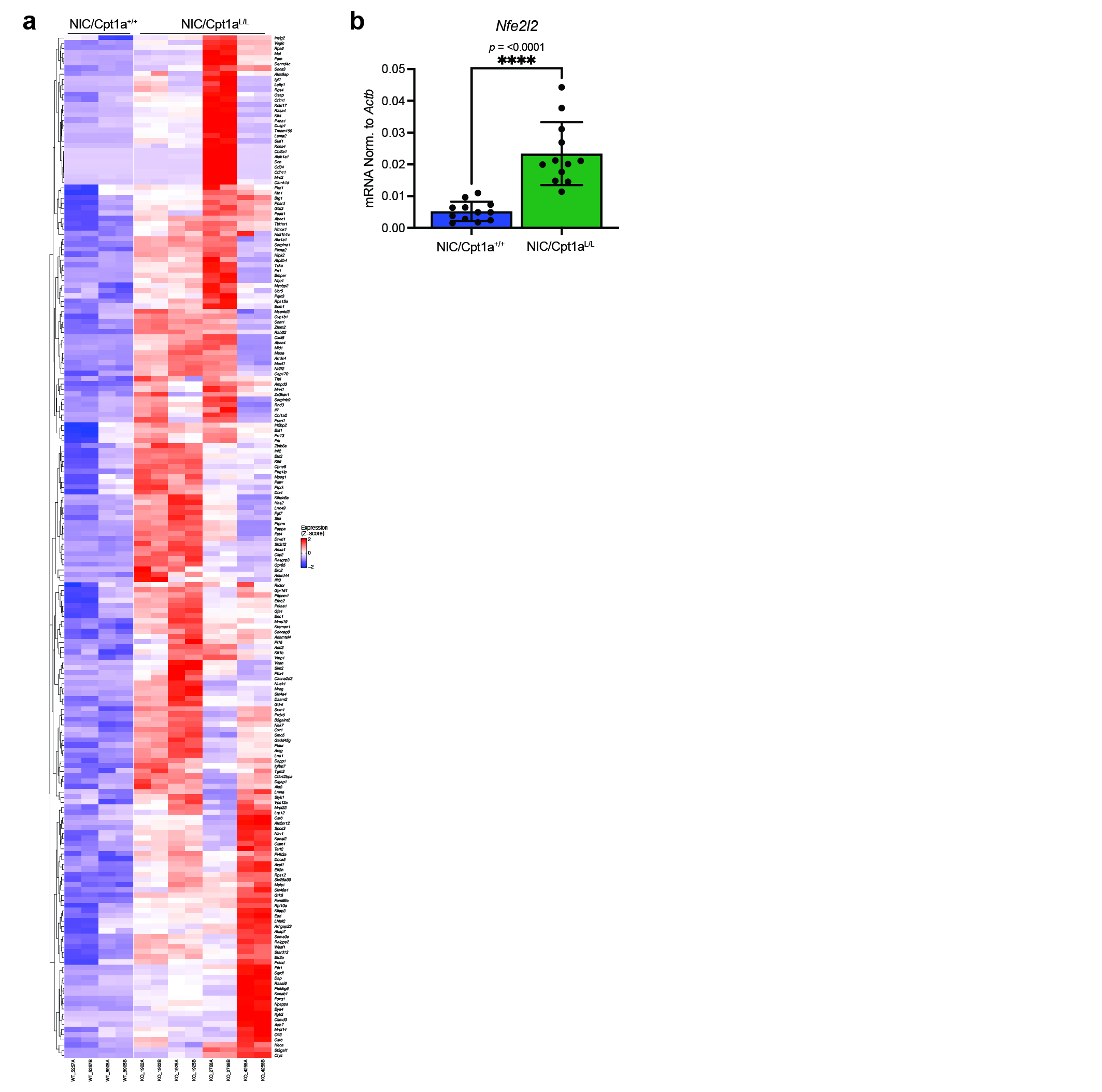
Supplementary Figure 6: Cpt1a deletion induces oxidative stress and elevates Nrf2 expression.**

**a,** Hierarchical clustering analysis of Nrf2 target gene expression (up-regulated (red) and down-regulated (blue)) in cell lines derived from NIC/Cpt1a^L/L^  tumors compared to NIC/Cpt1a^+/+^ controls (*n* = 2 or 4 per genotype, analyzed in duplicate). **b,** *Nrf2* mRNA levels in cell lines as in (a) were determined using QRT-PCR and normalized to *Actb*. *n* = 4 per genotype, analyzed in triplicate - *****p* < 0.0001; unpaired, two-tailed Student’s t-test. Error bars are expressed as mean values ± SD. Source data are provided as a Source Data file.

**
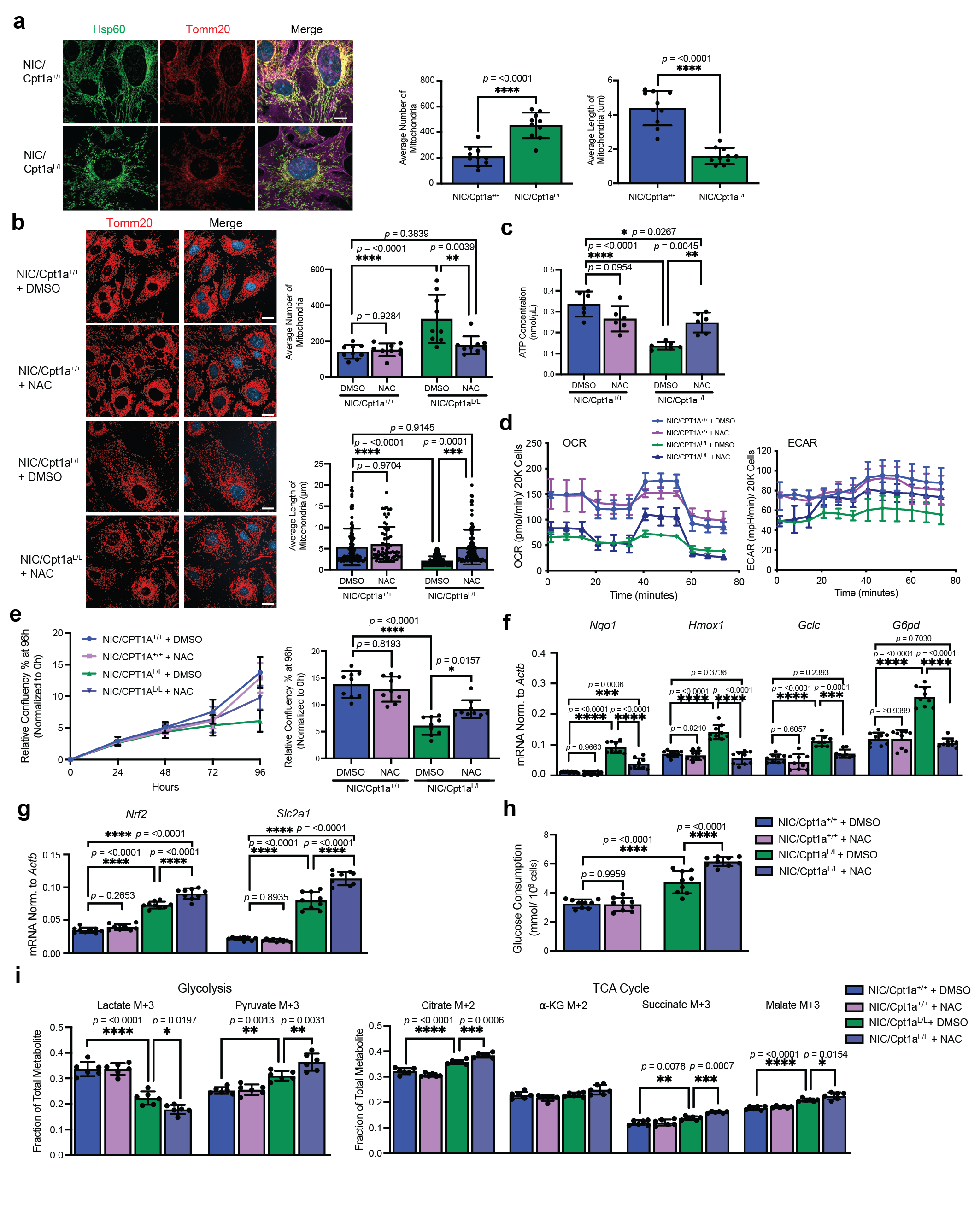
Supplementary Figure 7: Loss of Cpt1a disrupts mitochondrial fusion and function which can be reversed by ROS inhibition.**

**a,** Left panel – Representative images of NIC/Cpt1a^+/+^ and NIC/Cpt1a^L/L^  cells stained with antibodies against Hsp60 and Tomm20. Scale bar represents 10 μm. Right Panel – Number and average length of mitochondria were measured by co-localization of Hsp60 and Tomm20 with ImageJ. *n* = 3 per genotype, analyzed in triplicate -*****p* < 0.0001; unpaired, two-tailed Student’s t-test. **b,** Left panel - NIC cells treated with 5M N-acetyl cysteine (NAC), were stained for Tomm20. Scale bar represents 10 μm. Right Panel – Tomm20 staining analysis as in (a). *n* = 3 cell lines per genotype in triplicate – ***p* < 0.01, ****p* < 0.001, *****p* < 0.0001; one-way ANOVA with Tukey’s post-hoc test. **c,** ATP concentration in NIC cells following treatment with 5M NAC. *n* = 2 cell lines per genotype in triplicate – **p* < 0.05, ***p* < 0.01, *****p* < 0.0001; one-way ANOVA with Tukey’s post-hoc test. **d,** Basal, maximal (FCCP), ATP-synthesis coupled (Oligomycin A), and non-mitochondrial (rotenone/antimycin A) oxygen consumption rate (OCR – left panel) and extracellular acidification rate (ECAR - right panel) of NIC cells treated with 5M NAC. Representative of *n* = 3 cell lines in triplicate. **e,** Left panel - Proliferation assay of cells as in (b-d). Right panel – Confluency at 96 hours, normalized to t=0. *n=*3 cell lines in triplicate - **p* < 0.05 and *****p* < 0.0001; one-way ANOVA with Tukey’s post-hoc test. **f-g,** QRT-PCR analysis of Nrf2 target genes (f) and *Nrf2* and *Glut*1 (g) expression in NIC cells treated with 5M NAC. Gene expression was normalized to *Actb*. *n* = 3 cell lines per genotype in triplicate - ****p*< 0.001, *****p* < 0.0001; unpaired, two-tailed Student’s t-test. **h,** Glucose consumption of NIC cells treated with NAC or DMSO. *n* = 3 cell lines per genotype in triplicate – *****p* < 0.0001; one-way ANOVA with Tukey’s post-hoc test. **i,** Fractional ion abundance of glycolytic and TCA cycle intermediates of U-^13^C-glucose-labeled NIC cells treated with 5M NAC or DMSO. *n* = 2 per genotype, analyzed in triplicate - **p <* 0.05, ***p* < 0.01, ****p* < 0.001, *****p* < 0.0001; one-way ANOVA with Tukey’s post hoc-test. All error bars are expressed as mean values ± SD. Source data are provided as a Source Data file.

**
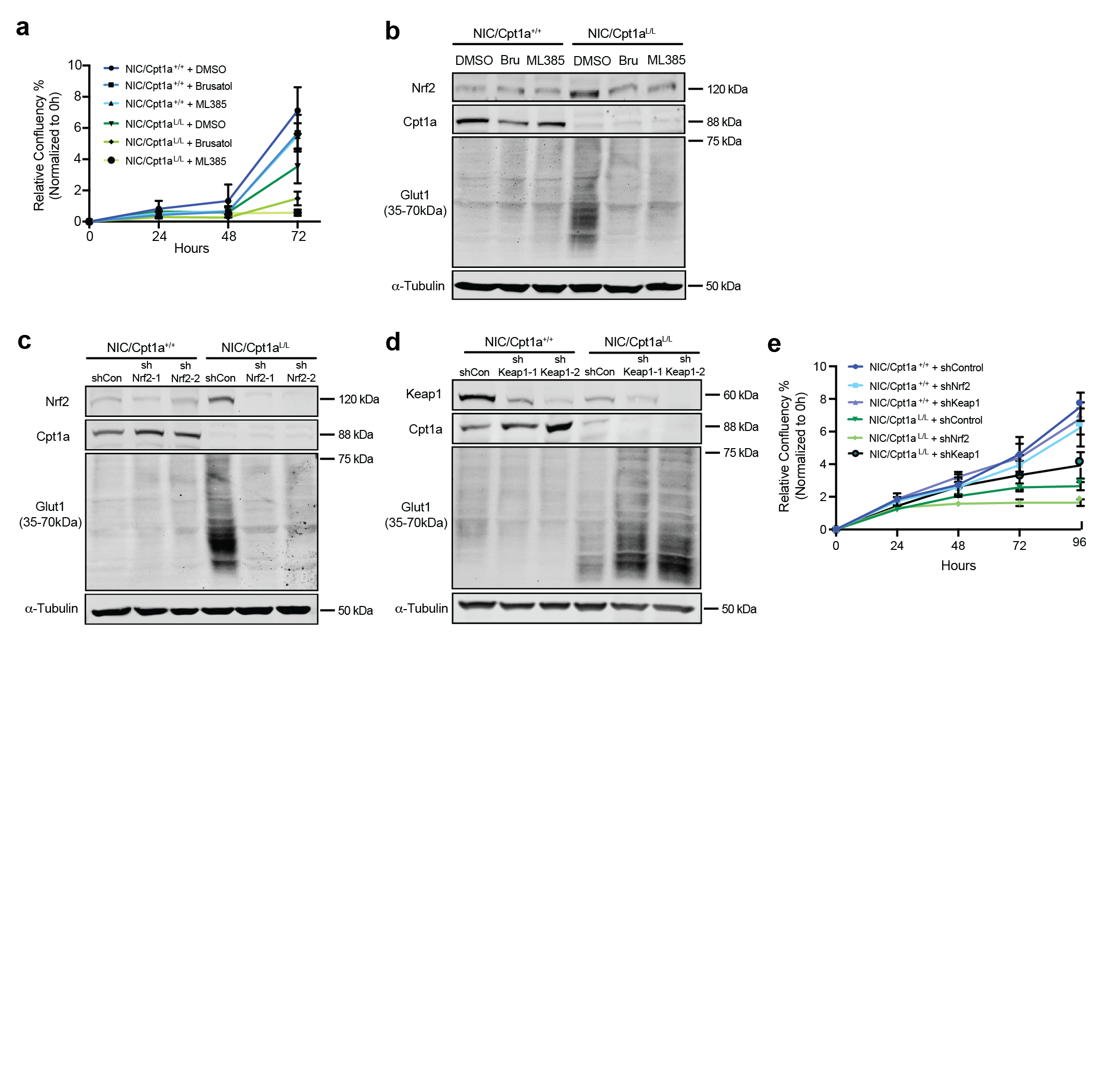
**

**Supplementary Figure 8: Nrf2 inhibition and genetic ablation blocks proliferation and glucose consumption of Cpt1a-deficient HER2+ breast cancer cells.**

**a,** NIC cells were treated with Nrf2 inhibitors (Brusatol and ML 385), or DMSO and proliferation was assessed in real time. Growth curves correspond to endpoint data shown in Figure 5a.  **b,**  Lysates of NIC/Cpt1a^+/+^ and NIC/Cpt1a^L/L^  cells were treated with Brusatol (100 nM), ML385 (2 μM) or DMSO and immunoblotted with the indicated antibodies. **c-d,** NIC cells stably expressing shRNAs against Nrf2, Keap1, or control (luciferase) were immunoblotted with the indicated antibodies **e,** Proliferation assay of NIC cells stably expressing shRNAs against Nrf2, Keap1, or control (luciferase), corresponding to the cells in Fig. 5f. All error bars are expressed as mean values ± SD. Source data are provided as a Source Data file.

**
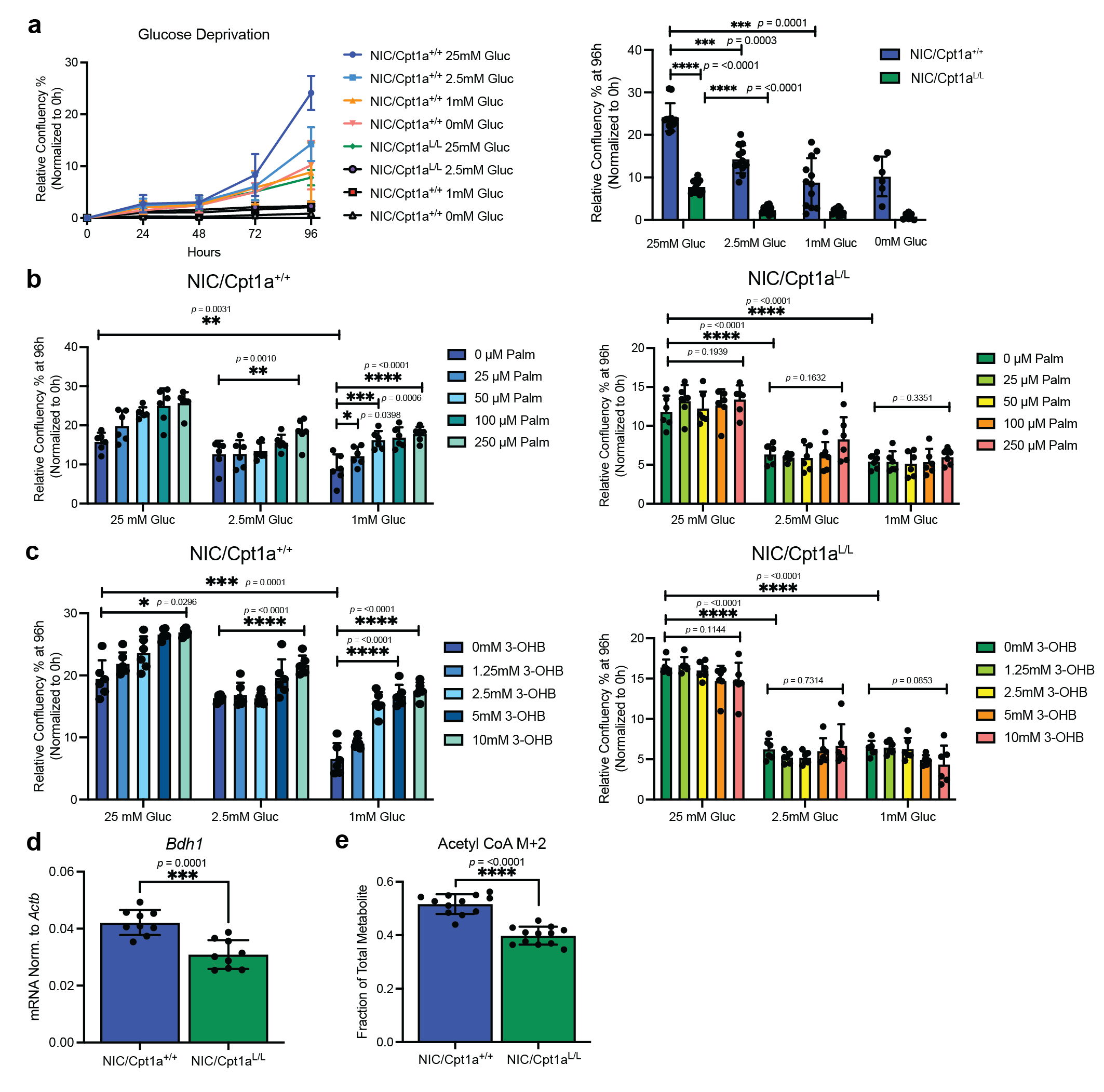
Supplementary Figure 9:** **Proliferation of Cpt1a-deficient cells is impaired in high-lipid or high-ketone, low-glucose conditions *in vitro***

**a,** Proliferation of NIC/Cpt1a^+/+^ and NIC/Cpt1a^L/L^  cells at the indicated glucose concentrations using an imaging-based assay to monitor cell confluency in real time. Left panel – growth curves. Right panel – endpoint analysis of cell growth at 96h. *n* = 2 cell lines in triplicate - ****p* < 0.001, *****p* < 0.0001; unpaired, two-tailed Student’s t-test. **b-c,** Proliferation of NIC cells supplemented with Palmitate (b) or Ketone (3-OHB, c) under varying glucose concentrations for 96 hours. Endpoint analysis after 96h of imaging is shown. Data were normalized to confluency at t=0. *n* = 3 cell lines per genotype in triplicate – **p* < 0.05, ***p* < 0.01, ****p* < 0.001, *****p* < 0.0001; one-way ANOVA with Tukey’s post-hoc test. **d,** QRT-PCR analysis of 3-betahydroxybutyrate dehydrogenase 1 (*Bdh1*) in NIC/ Cpt1a^+/+^ and NIC/ Cpt1a^L/L^ cells. Expression was normalized to *Actb*. *n* = 3 cell lines per genotype in triplicate - ****p*< 0.001, unpaired, two-tailed Student’s t-test. **e,** Fractional ion abundance of Acetyl CoA (M+2) in NIC/ Cpt1a^+/+^ and NIC/ Cpt1a^L/L^  cells following a 2 hour pulse with U-^13^C-glucose. *n* = 3 per genotype, analyzed in triplicate - ****p* < 0.001; unpaired, two-tailed Student’s t-test. All error bars are expressed as mean values ± SD. Source data are provided as a Source Data file.

**
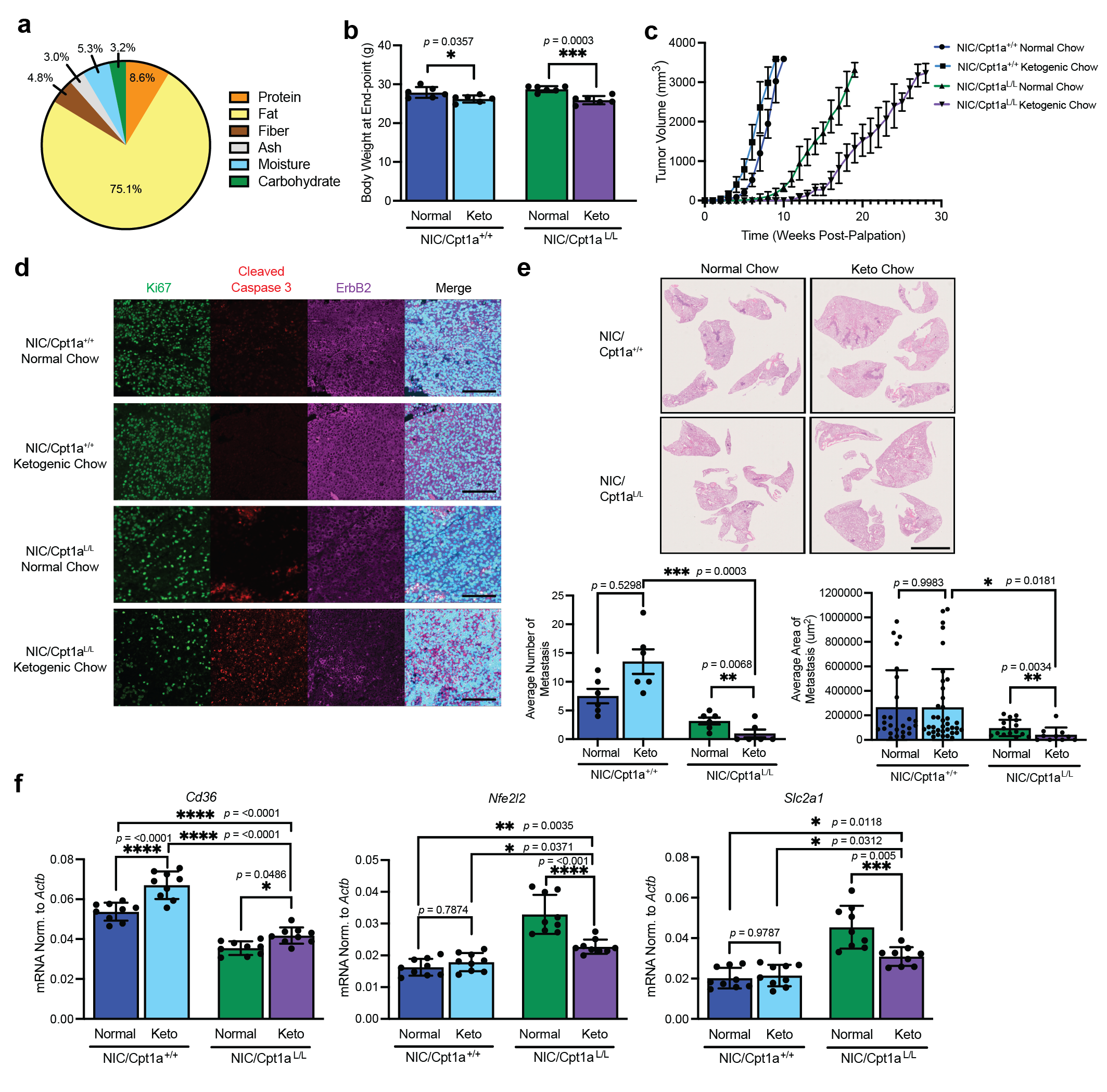
Supplementary Figure 10: Loss of Cpt in combination with the ketogenic diet decreases tumor growth and metastasis.**

**a,** Ingredient composition of normal chow and ketogenic diet chow fed to female FVB/N for 15 weeks. **b,** Mouse body weight was measured to ensure mice achieved ketosis. *n* = 6 per group, *****p* < 0.0001; one-way ANOVA with Tukey’s post-hoc test**. c,** Tumor burden was determined by weekly caliper measurements till all mice reached end-point. *n* = 6 mice per treatment group. **d,** End-stage tumors from FVB/N mice bearing orthotopic allografts of NIC/Cpt1a^+/+^ and NIC/Cpt1a^L/L^  cells fed with normal or ketogenic chow were immunostained using the indicated antibodies and DAPI. Images are representative of 6 mice per treatment group. Scale bar: 100 μm. **e,** Top panel – representative H&E images of lungs from mice as in (A). Scale bar: 5 mm. Bottom panels – average number and total area of lung metastases. *n* = 6 per treatment group, **p* < 0.05, ***p* < 0.01 and ****p* < 0.001; one-way ANOVA with Tukey’s post-hoc test. **f,** QRT-PCR analysis of *Cd36*, *Nrf2* and *Glut1* gene expression in tumors samples as in (a). Gene expression was normalized to that of *Actb*. *n* = 6 per treatment group. **p <* 0.05, ***p* < 0.01, ****p* < 0.001, and *****p* < 0.0001, by one-way ANOVA with Tukey’s post hoc test. All error bars are expressed as mean values ± SD. Source data are provided as a Source Data file.

**
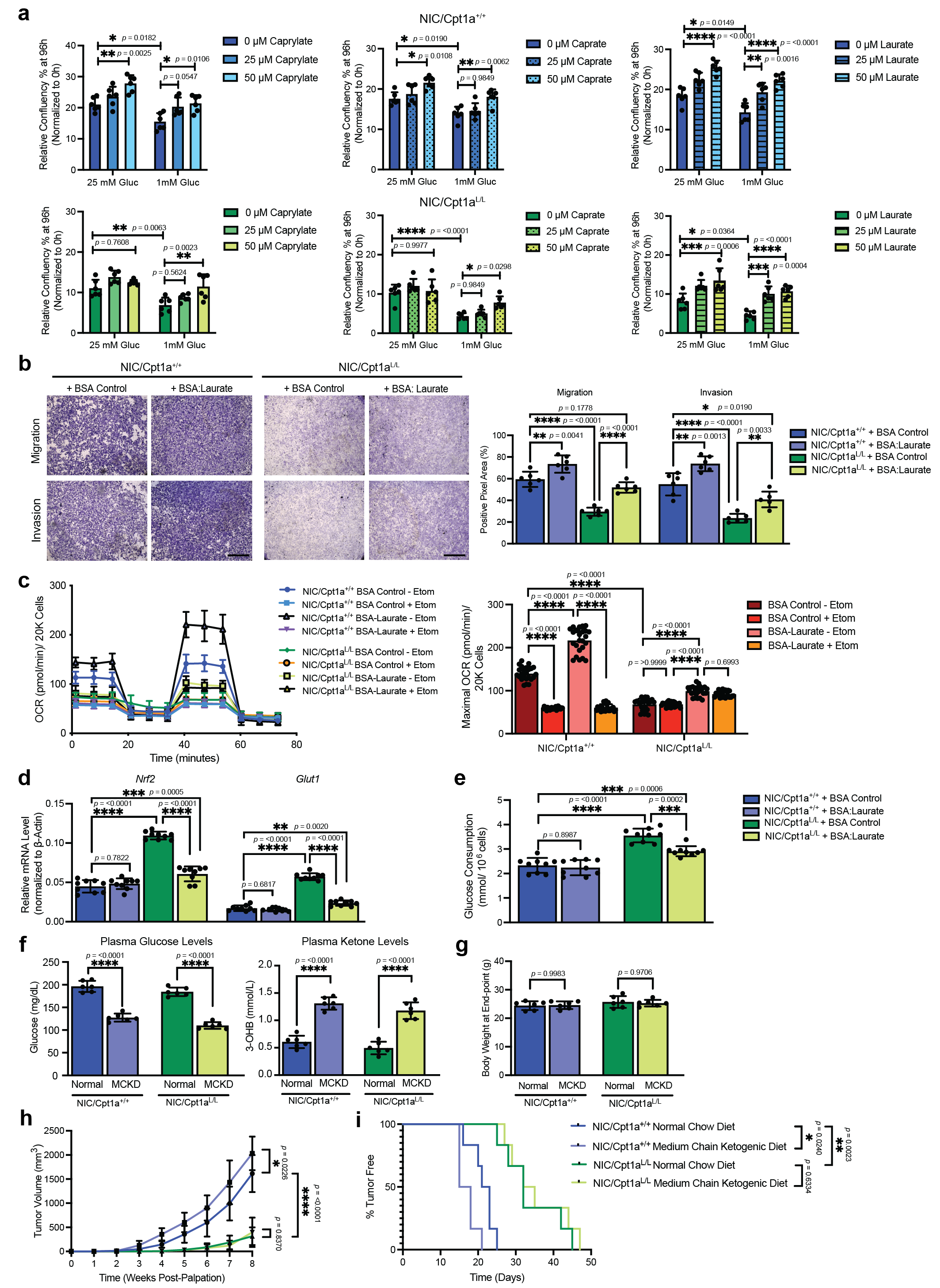
Supplementary Figure 11: Supplementation with medium-chain fatty acids partially rescues proliferation and respiration of Cpt1a-deficient NIC cells *in vitro.***

**a,** Proliferation assessed on Cpt1a-proficient (top panels) and -deficient (bottom panels) NIC cells supplemented with caprylate (C8:0), caprate (C10:0) and laurate (C12:0) under varying glucose concentrations for 96 hours. Data normalized to confluency at t=0. *n* = 2 cell lines per genotype in triplicate – **p* < 0.05, ***p* < 0.01, ****p* < 0.001, *****p* < 0.0001; one-way ANOVA with Tukey’s post-hoc test. **b,** Left panel – Representative images of cell migration and invasion (Boyden chamber) assays of NIC cells supplemented with 50 μM BSA-Laurate or BSA control. Scale bar represents 1000 μm. Right Panel – quantification (positive pixel area) of migration and invasion (n = 3 cell lines per genotype in triplicate – **p* < 0.05, ***p* < 0.01, *****p* < 0.0001; one-way ANOVA with Tukey’s post-hoc test). **c,** Left panel - Basal, maximal (FCCP), ATP-synthesis coupled (Oligomycin A), and non-mitochondrial (rotenone/ antimycin A) oxygen consumption rates (OCRs) of NIC cells with BSA-control or Laurate, and in the presence or absence of 10μM Etomoxir (Etom). Right panel - Quantification of maximal OCR. *n* = 2 cell lines in quadruplicate - *****p* < 0.0001; one-way ANOVA with Tukey’s post-hoc test. **d-e,** QRT-PCR analysis of *Nrf2* and *Glut1* gene expression (d) and Glucose consumption (e) of cells as in (b-c) treated with 50 μM BSA-Laurate or BSA. *n* = 3 cell lines per genotype in triplicate – ***p* < 0.01, ****p* < 0.001, *****p* < 0.0001; one-way ANOVA with Tukey’s post-hoc test. **f,** Medium-chain ketogenic diet (MCKD) orthotopic xenotransplant study using two independent NIC/Cpt1a^+/+^ and NIC/Cpt1a^L/L^  cell lines. Plasma glucose and ketone levels were assessed. *n* = 6 mice per group, *****p* < 0.0001; one-way ANOVA with Tukey’s post-hoc test. **g,** Mouse body weight at tumor end-point. *n* = 6 mice per treatment group; one-way ANOVA with Tukey’s post-hoc test. **h,** Tumor burden determined by weekly caliper measurements. *n* = 6 mice per treatment group, **p <* 0.05, *****p* < 0.0001; one-way ANOVA with Tukey’s post-hoc test. **i,** Kaplan-Meier survival analysis. *n* = 6 mice per treatment group, **p* < 0.05, ***p* < 0.01; log rank test. All error bars are expressed as mean values ± SD. Source data are provided as a Source Data file.

**
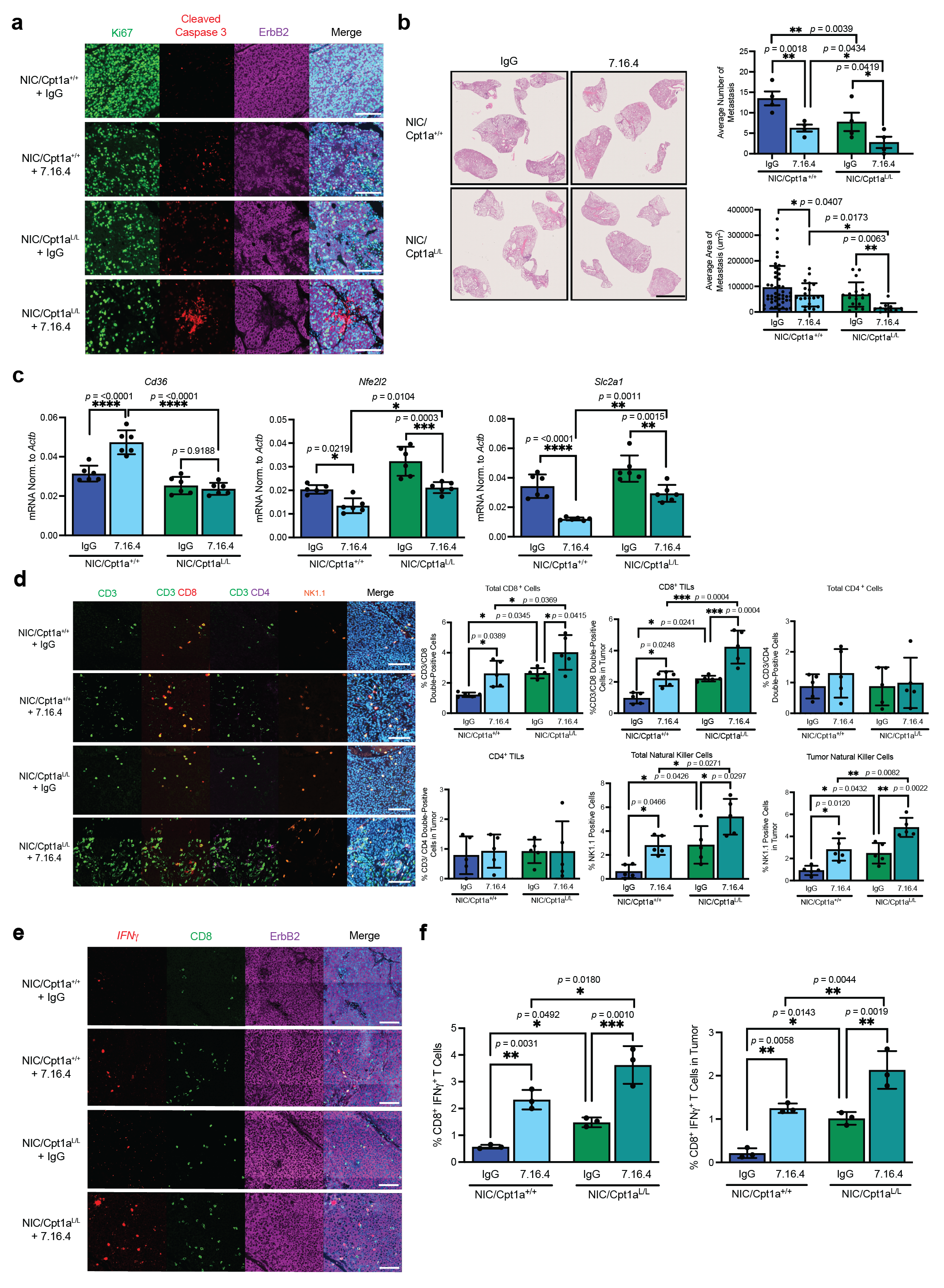
Supplementary Figure 12: CPT1A deletion enhances the effects of ErbB2 monoclonal antibody therapy to dampen tumor progression in ErbB2+ breast cancer cells.**

**a,** End-stage tumors from immunocompetent mice bearing orthotopic allografts of NIC/ Cpt1a^+/+^ and NIC/ Cpt1a^L/L^ cells treated with IgG (control Ab) and 7.16.4 Ab were immunostained using the indicated antibodies and DAPI. Images are representative of 6 mice per treatment group. Scale bar: 100 μm. **b,** Left panel – representative H&E images of lungs from mice as in (a). Right panels – average number and total area of lung metastases. *n* = 10 per treatment group, **p* < 0.05 and ***p* < 0.01; one-way ANOVA with Tukey’s post-hoc test. **c,** QRT-PCR analysis of *Cd36, Nrf2* and *Glut1* gene expression in tumors samples as in (A). Gene expression was normalized to that of *Actb*. *n* = 6 per treatment group. **p <* 0.05, ***p* < 0.01, ****p*< 0.001, and *****p* < 0.0001, by one-way ANOVA with Tukey’s post hoc test. **d,** Left panel - End-stage tumors from mice bearing orthotopic allografts of NIC/ Cpt1a^+/+^ and NIC/ Cpt1a^L/L^ cells treated with IgG and 7.16.4 Ab were immunostained using the indicated markers of immune cells and DAPI. Images are representative of 5 mice per treatment group. Scale bar: 100 μm. Right panel - Quantification of CD3^+^/CD8^+^ (total and tumor), CD3^+^/CD4^+^ (total and tumor), and NK1.1 (total and tumor) by HALO Analysis. **p* < 0.05, ***p <* 0.01, ****p* < 0.001; one-way ANOVA with Tukey’s post hoc-test. **e,** RNA FISH staining against IFNγ (red dots) combined with immunofluorescence staining for CD8, ErbB2 and DAPI on tumor tissue from NIC/ Cpt1a^+/+^ and NIC/ Cpt1a^L/L^ mice treated with IgG and 7.16.4 Ab. Images are representative of 3 mice per treatment group. Scale bar: 100 μm. **f,** Quantification of total CD8^+^ IFNγ^+^ cells (IFNγ-secreting cytotoxic T cells) (left panel) and tumor-infiltrating CD8^+^ IFNγ^+^ (CD8^+^ IFNγ^+^ ErbB2^+^) (right panel) in NIC/ Cpt1a^+/+^ and NIC/ Cpt1a^L/L^ tumors after treatment with IgG and 7.16.4 Ab. *n* = 3 per treatment group, **p* < 0.05, ***p* < 0.01 and ****p* < 0.001; one-way ANOVA with Tukey’s post-hoc test. All error bars are expressed as mean values ± SD. Source data are provided as a Source Data file.

**Supplementary Tables**

Supplementary Table S1: Genotyping Primers

| **Gene** | **Sequences** | **Species** |
| --- | --- | --- |
| Neu Genotyping Forward Primer | 5’-TTCCGGAACCCACATCAGGCC-3’ | Mouse |
| Neu Genotyping Reverse Primer | 5’-GTTTCCTGCAGCAGCCTACGC-3’ | Mouse |
| Cre Genotyping Forward Primer | 5’-TGCTCTGTCCGTTTGCCG-3’ | Mouse |
| Cre Genotyping Reverse Primer | 5’-ACTGTGTCCAGACCAGGC-3’ | Mouse |
| Flox Cpt1a Genotyping Forward Primer | 5’-TGAAGGTGCTGCTCTCCTACCATGG-3’ | Mouse |
| Flox Cpt1a Genotyping Reverse Primer | 5’-CATGGTGCCTTGGCCTTAAGGTC-3’ | Mouse |

Supplementary Table S2: Dietary Composition of Normal and Ketogenic Diets

|  | **Normal Chow** | **LCKD** | **MCKD** |
| --- | --- | --- | --- |
| **Product Number** | Teklad, 2920X | Bioserv, F3666 | Bioserv, F10595 |
| **Macronutrient** |  | | |
| Protein | 19.4 % | 8.6 % | 8.6 % |
| Fat | 6.5 % | 75.1 % | 75.1 % |
| Carbohydrate | 47.0 % | 3.2 % | 3.2 % |
| Crude Fiber | 2.7 % | 4.8 % | 4.8 % |
| Ash | 5.1 % | 3.0 % | 3.0 % |
| **Caloric Profile** |  | | |
| Calories from Protein | 24.0 % | 4.7 % | 4.7 % |
| Calories from Fat | 16.0 % | 93.3 % | 93.3 % |
| Calories from Carbohydrate | 60.0 % | 1.8 % | 1.8 % |
| Energy Density | 3.10 kcal/g | 7.24 kcal/g | 7.24 kcal/g |
| **Fatty Acids (% of Total Fatty Acids)** |  | | |
| C18:2ω6 Linoleic | 2.6 % | 10.3 % | 3.3 % |
| C18:3ω3 Linolenic | 0.3 % | 0.6 % | 0.3 % |
| Total Saturated | 0.8 % | 27.3 % | 63.1 % |
| Total Monounsaturated | 1.1 % | 25.9 % | 5.0 % |
| Total Polyunsaturated | 2.9 % | 11.0 % | 3.5 % |

Supplementary Table S3: Primary and Secondary Antibodies

| **Antibody** | **Source** | **Catalog #** | **Dilution** |
| --- | --- | --- | --- |
| β-Actin | Millipore | A5441 | IB: 1/2000 |
| CD206/ MRC1 | Cell Signaling | 24595 | IF: 1/400 |
| CD3-epsilon | Cell Signaling | 99940 | IF: 1/200 |
| CD36 | Novus Biologicals | NB400-144 | IF: 1/200 |
| CD4 | Cell Signaling | 25229 | IF: 1/200 |
| CD8-alpha | Cell Signaling | 98941 | IF: 1/200 |
| CD31 (PECAM-1) | Cell Signaling | 77699 | IF: 1/200 |
| Cleaved Caspase 3 | Cell Signaling | 9661 | IF: 1/200 |
| CPT1A | Proteintech | 15184-1-AP | IF: 1/500  IB: 1/1000 |
| ErbB2 | DAKO | A0485 | IF: 1/200 |
| ErbB2/ c-Neu (Ab3) | Millipore | OP15L | IF: 1/100 |
| F4/80 | Cell Signaling | 70076 | IF: 1/200 |
| Glut1 | Cell Signaling | 73015 | IF: 1/200  IB: 1/1000 |
| Hsp60 | Fisher Scientific | MA5-15836 | IF: 1/100 |
| Keap1 | Proteintech | 10503-2-AP | IB: 1/1000 |
| Ki67 | Cell Signaling | 12202 | IF: 1/200 |
| Mpc2 | Cell Signaling | 46141 | IB: 1/1000 |
| NK1.1/ CD161 | Cell Signaling | 39197 | IF: 1/200 |
| Nrf2 | Cell Signaling | 12721 | IF: 1/200  IB: 1/1000 |
| p-Stat1 (Y701) | Cell Signaling | 9167 | IF: 1/200 |
| Tomm20 | Santa Cruz | sc-11415 | IF: 1/200 |
| α-Tubulin | Cell Signaling | 3873 | IB: 1/2000 |
| Vinculin | Chemicon | MAB3574 | IB: 1/5000 |
| Alexa Fluor 488 Goat anti-Mouse | Fisher Scientific | A32723 | IF: 1/1000 |
| Alexa Fluor 555 Goat anti-Rabbit | Fisher Scientific | A32732 | IF: 1/1000 |
| Alexa Fluor 647 Phalloidin | Fisher Scientific | A22287 | IF: 1/1000 |
| IRDye 800CW Donkey anti-Rabbit | Li-COR Biosciences | 925-32213 | IB: 1/10000 |
| IRDye 680RD Donkey anti-Mouse | Li-COR Biosciences | 926-68073 | IB: 1/10000 |

Supplementary Table S4: Mouse quantitative RT-PCR primer sequences

| **Gene** | **Sequences** | **Species** |
| --- | --- | --- |
| *Nfe2l2* qPCR Forward Primer | 5’-TCTTGGAGTAAGTCGAGAAGTGT-3’ | Mouse |
| *Nfe2l2* qPCR Reverse Primer | 5’-GTTGAAACTGAGCGAAAAAGGC-3’ | Mouse |
| *Slc2a1* qPCR Forward Primer | 5’-CAGTTCGGCTATAACACTGGTG-3’ | Mouse |
| *Slc2a1* qPCR Reverse Primer | 5’-GCCCCCGACAGAGAAGATG-3’ | Mouse |
| *Slc2a2* qPCR Forward Primer | 5’-TCAGAAGACAAGATCACCGGA-3’ | Mouse |
| *Slc2a2* qPCR Reverse Primer | 5’-GCTGGTGTGACTGTAAGTGGG-3’ | Mouse |
| *Slc2a3* qPCR Forward Primer | 5’-ATGGGGACAACGAAGGTGAC-3’ | Mouse |
| *Slc2a3* qPCR Reverse Primer | 5’-GTCTCAGGTGCATTGATGACTC-3’ | Mouse |
| *Slc2a4* qPCR Forward Primer | 5’GTGACTGGAACACTGGTCCTA-3’ | Mouse |
| *Slc2a4* qPCR Reverse Primer | 5’-CCAGCCACGTTGCATTGTAG-3’ | Mouse |
| *Cd36* qPCR Forward Primer | 5’-ATGGGCTGTGATCGGAACTG-3’ | Mouse |
| *Cd36* qPCR Reverse Primer | 5’-GTCTTCCCAATAAGCATGTCTCC-3’ | Mouse |
| *Bdh1* qPCR Forward Primer | 5’- ACAAGACACACGCTGTTGTTT-3’ | Mouse |
| *Bdh1* qPCR Reverse Primer | 5’- CTCTTCAAGCTGTCCAGTTC-3’ | Mouse |
| *Nqo1* qPCR Forward Primer | 5’-AGGATGGGAGGTACTCGAATC-3’ | Mouse |
| *Nqo1* qPCR Reverse Primer | 5’-AGGCGTCCTTCCTTATATGCTA-3' | Mouse |
| *G6pd* qPCR Forward Primer | 5’-CACAGTGGACGACATCCGAAA-3’ | Mouse |
| *G6pd* qPCR Reverse Primer | 5’-AGCTACATAGGAATTACGGGCAA-3’ | Mouse |
| *Hmox1* qPCR Forward Primer | 5’-AAGCCGAGAATGCTGAGTTCA-3' | Mouse |
| *Hmox1* qPCR Reverse Primer | 5’-GCCGTGTAGATATGGTACAAGGA-3’ | Mouse |
| *Gclc* qPCR Forward Primer | 5’-GGGGTGACGAGGTGGAGTA-3’ | Mouse |
| *Gclc* qPCR Reverse Primer | 5’- GTTGGGGTTTGTCCTCTCCC-3’ | Mouse |
| *Actb* qPCR Forward Primer | 5’- TCCATCATGAAGTGTGACGT-3’ | Mouse |
| *Actb* qPCR Reverse Primer | 5’- GAGCAATGATCTTGATCTTCAT-3’ | Mouse |
