# Supplementary material for: Targeting fatty acid oxidation enhances response to HER2-targeted therapy
Source: Nat Commun. 2024 Aug 3;15:6587. doi: 10.1038/s41467-024-50998-3 (PMC11297952; doi:10.1038/s41467-024-50998-3)
Supplement: Supplementary file 3 — Reporting Summary [file 41467_2024_50998_MOESM3_ESM.pdf]

nature portfolio

Corresponding author(s): William J. Muller

Last updated by author(s): Jul 19, 2024

Reporting Summary

Nature Portfolio wishes to improve the reproducibility of the work that we publish. This form provides structure for consistency and transparency in reporting. For further information on Nature Portfolio policies, see our Editorial Policies and the Editorial Policy Checklist.

Statistics

For all statistical analyses, confirm that the following items are present in the figure legend, table legend, main text, or Methods section.

n/a	Confirmed	
☐	☒	The exact sample size (n) for each experimental group/condition, given as a discrete number and unit of measurement
☐	☒	A statement on whether measurements were taken from distinct samples or whether the same sample was measured repeatedly
☐	☒	The statistical test(s) used AND whether they are one- or two-sided<br>*Only common tests should be described solely by name; describe more complex techniques in the Methods section.*
☒	☐	A description of all covariates tested
☐	☒	A description of any assumptions or corrections, such as tests of normality and adjustment for multiple comparisons
☐	☒	A full description of the statistical parameters including central tendency (e.g. means) or other basic estimates (e.g. regression coefficient) AND variation (e.g. standard deviation) or associated estimates of uncertainty (e.g. confidence intervals)
☐	☒	For null hypothesis testing, the test statistic (e.g. F, t, r) with confidence intervals, effect sizes, degrees of freedom and P value noted<br>*Give P values as exact values whenever suitable.*
☒	☐	For Bayesian analysis, information on the choice of priors and Markov chain Monte Carlo settings
☒	☐	For hierarchical and complex designs, identification of the appropriate level for tests and full reporting of outcomes
☒	☐	Estimates of effect sizes (e.g. Cohen's d, Pearson's r), indicating how they were calculated

Our web collection on statistics for biologists contains articles on many of the points above.

Software and code

Policy information about availability of computer code

Data collection

RNA-Seq: RNA was isolated from two (NIC/Cpt1a+/+) or four (NIC/Cpt1aL/L) independent tumor-derived cell lines in duplicate, and quality was assessed using a Nanodrop 2000 (Thermo Fisher Scientific, ND2000CLAPTOP). RNA was sequenced and analyzed by Novogene. Briefly, the reference genome mm10 (mouse) index was obtained from NCBI/ UCSC/ Ensembl. Clean reads were processed by taking the raw reads and removing reads containing adaptors, with more than 0.1% of undetermined bases and were of low quality. Using STAR (v2.5), clean reads were mapped directly to the reference genome. Read counts were performed using HTSeq v0.6.1 and transcript abundance was determined using Fragments per kilobase of transcript sequence per million base pairs sequenced (FPKM) which accounts for sequencing depth and gene length. Differential expression analysis was performed using the DEGseq2 R package (2_1.6.3) with p-values adjusted using Benjamini and Hochberg's method. Genes with adjusted p-values under 0.05 were assigned as differentially expressed. FPKM levels were utilized to evaluate correlation differences and plotted using unsupervised hierarchal clustering, self-organization mapping (SOM) and kmeans. Analysis of transcriptional regulation in differentially expressed genes was performed using Enrichr.

Metabolomics and Gas Chromatography/Mass Spectrometry analysis

Metabolic profiling and isotope tracing analyses using 13C-glucose and 13C-palmitate were performed at the Metabolomics Innovation Resource, McGill University. Briefly, Cpt1a-proficient and -deficient NIC cells (n = 3 cell lines per genotype, analyzed in triplicate) were cultured in unlabelled DMEM supplemented with 2% dialyzed FBS (Wisent, 080-910) in 6-cm dishes (Nunc) for 48 hours. The media was then replaced with glucose-free DMEM supplemented with 2% dialyzed FBS and 25mM [U-13C]-glucose (Cambridge Isotope Laboratories, CLM-1396, 99% atom 13C) for either 30 minutes or 2 hours, or [U-13C]-palmitate (Cambridge Isotope Laboratories, CLM-409, 99% atom 13C) for 24 hours. Additionally, some dishes were kept in unlabeled media as controls. Cells were washed three times in ice-cold saline solution (NaCl, 0.9g/L), and water-soluble metabolites were extracted in 80% methanol (GC/MS grade). After two 10-minute rounds of sonication (30 seconds on/ 30 seconds off at high intensity) on slurry ice using the Bioruptor UCD-200 sonicator, the homogenates were centrifuged at 15,000 ×g for 10 minutes at 4C. Supernatants were collected and an internal standard, 800 ng Myristic acid-D27, was added to each sample. Samples dried

by vacuum centrifugation (CentriVap Concentrator; Labconco, KS, USA) overnight at -1°C were resuspended in 30µl of 10 mg/mL methoxyamine hydrochloride in anhydrous pyridine and incubated for 30 minutes at room temperature. Samples were then transferred to GC–MS autoinjector vials containing 70µl N-(tert-butyldimethylsilyl)-N-methyltrifluoroacetamide (MTBSTFA) derivatization reagent and incubated at 70°C for 1hour. A blank sample, composed of 30µl of 10 mg/mL methoxyamine-HCl pyridine and 70µl of MTBSTFA, was also prepared. A volume of 1µl of sample was injected splitless with an inlet temperature of 280°C into the GC-MS instrument, Agilent 5975C. Metabolites were resolved by separation on a DB-5MS + DG (30m x 250 µm x 0.25 µm) capillary column (Agilent Technologies, CA, USA). Helium was used as the carrier gas with a flow rate such that Myristic-D27 acid eluted at approximately 18 minutes. The quadrupole was set at 150°C, the source at 230°C, and the GC/MS interface at 320°C. The oven program started at 60°C, held for 1 minute, then increased at a rate of 10°C per minute until 320°C. Bake-out was at 320°C for 9 minutes. Metabolites were ionized by electron impact at 70eV. All samples were injected using scan (50-1000m/z) and selected ion monitoring (SIM) mode. In all experiments, Cpt1a-proficient NIC cells were used as controls. The sample preparation and data collection order for biological and technical replicates was randomized. All metabolites described in this study were validated against authenticated standards to confirm mass spectra and retention times. The relative amount of each metabolite was determined from the integration of ion intensities and normalized to the number of cells extracted using MassHunter Quant software (Version B.08.00) (Agilent Technologies) according to published protocols. Mass isotopomer distribution analysis was determined using a custom in-house algorithm developed at McGill University (McGuirk).

BioNova: Glucose and lactate levels in conditioned media were measured using a Flux Bioanalyzer (NOVA Biomedical) according to the manufacturer's instructions.

Respirometry and extracellular acidification measurements: Oxygen consumption and extracellular acidification rates were measured using an XFe96 Extracellular Flux Analyzer (Seahorse Bioscience) using the manufacturer's established protocols. At the end of the assay, the IncuCyte S3 system (ESSEN BioSciences, Ann Arbor, MI, USA) was used for live cell counting at 10x magnification. Cell counts were determined using the IncuCyte S3 Analysis software. Data was normalized to cell number (20,000 cells) and analyzed using Wave software (Seahorse Bioscience).

Lung metastasis: Three H&E stained 10um step sections per sample were scanned using an Aperio-XT slide scanner (Leica Biosystems) and analyzed using Imagescope software (Leica Biosystems).

IF, IHF and RNA FISH: Mitochondrial Immunofluorescence Images and Tumor Immunohistofluorescence images were acquired using a Zeiss LSM800 confocal microscope or Zeiss Axioscan slide scanner. For mitochondrial morphology analysis, images from cells labeled for HSP60 and TOMM20 were obtained using the 60X objective and stacked in the same condition of gain, laser intensities and exposure time. Images were then compiled as "Max projection" and analyzed using the ImageJ/FIJI software (v1.53u, NIH).

RT-PCR: Total RNA was extracted from cultured cells or flash-frozen mammary tumors using the RNeasy Mini Kit (Qiagen, 74106). mRNA was reverse-transcribed into cDNA using the ProtoScript First Strand cDNA Synthesis Kit (New England Biolabs, E6300). Real-time quantitative PCR (qRT-PCR) was performed using the LightCycler 480 SYBR Green 1 MasterMix (Roche, 04887352001), run on the LightCycler 480 instrument (Roche) and analyzed using the corresponding software (LightCycler 480 SW, v1.5.1.62). Each sample was run in triplicate and normalized to Actb as a control. qRT-PCR Primer sequences are in Supplementary Table S4.

Immunoblots: Immunoblot images were acquired using a Li-COR Odyssey system and analyzed on the associated software, Image Studio Lite (v5.2.1, Li-COR Biosciences).

In vitro Migration and Invasion Assay: Images were acquired using an AxioZoom V16 (Zeiss) and analyzed (positive pixel) using ImageJ (v1.53u, NIH).

ROS Detection: Images were acquired using an EVOS FL Microscope (Thermo Fischer Scientific, 4471136) and analyzed (positive pixel) using ImageJ (v1.53u, NIH).

Neutral lipid staining and uptake of BODIPY using flow cytometry: Cells were treated with 2 uM of cell membrane-permeant fluorophore, BODIPY 493/503 (4,4-Difluoro-1,3,5,7,8-Pentamethyl-4-Bora-3a,4a-Diaza-s¬-Indacene) (Thermo Fischer Scientific, #D3922) or BODIPY FL C16 (4,4-Difluoro-5,7-Dimethyl-4-Bora-3a,4a-Diaza-s-Indacene-3-Hexadecanoic Acid) (Thermo Fischer Scientific, #D3821) for 30 minutes at 37 C to stain for lipid droplets. Samples were washed with PBS to remove staining solution, trypsinized to generate single cell suspension and centrifuged at 800 ×g for 3 minutes. Subsequently, cells were washed with FACS buffer (1X PBS with 2 mM EDTA and 2% FBS), passed through a 70m strainer and stained with viability dye eFluorYM 506 (eBioscience, 65-00866-18) for 10 minutes on ice. A minimum of 100,000 events per sample was acquired using a LSR Fortessa 5L Flow Cytometer (BD Bioscience, H649225B4076) with FACSDiva Software (Version 8) (BD Biosciences) in slow rate mode to avoid doublets. Cell populations were gated as shown in Supplementary Fig. 2d. Data were analyzed with FlowJo Software (Ashland, OR, USA). Cell debris and aggregates were excluded from the analysis using pulse processing SSC-H vs SSC-W.

Incucyte Cell Proliferation Assays: 5000 cells per well were seeded in triplicate or quadruplicate in 96-well optical-bottom plates (Nunc, 167008). After 24 hours of seeding, drugs or vehicle controls were added, and live cell imaging was performed using the IncuCyte S3 system (ESSEN BioSciences, Ann Arbor, MI, USA) at 10x magnification every 6 hours over a period of 96 hours (2 images per well per timepoint, 17 total timepoints). Percentage confluence was determined using the IncuCyte S3 Analysis software (v2019A, ESSEN BioSceinces).

Data analysis	Statistical Analysis was performed on Prism 9.0 (Broad Institute, v4.i0), ImageJ (v1.53u, NIH), BioRender (https://www.biorender.com/), Kaplan-Meier survival curves (https://kmplot.com/analysis/), ROC Plot (ROCPlot.org), Enrichr (Ma'yaan Labs, https://maayanlab.cloud/Enrichr/), HALO Software Image Analysis (v3.5.3577, Indica Labs), Seahorse Wave (v2.6.0.31, Agilent), Adobe Illustrator (v25.2.3), Image Studio Lite (v5.2.1, Li-COR Biosciences), Microsoft Excel (v16.66.1, Microsoft), IncuCyte S3 system (v2019A, ESSEN BioSciences), FACSDiva Software (Version 8) (BD Biosciences), MassHunter Quant (v12.0.893.1, Agilent) and FlowJo Software (v10.10_CL, Ashland, OR, USA). More detail is provided in the manuscript.

For manuscripts utilizing custom algorithms or software that are central to the research but not yet described in published literature, software must be made available to editors and reviewers. We strongly encourage code deposition in a community repository (e.g. GitHub). See the Nature Portfolio guidelines for submitting code & software for further information.

Data

Policy information about availability of data

All manuscripts must include a data availability statement. This statement should provide the following information, where applicable:

- Accession codes, unique identifiers, or web links for publicly available datasets
- A description of any restrictions on data availability
- For clinical datasets or third party data, please ensure that the statement adheres to our policy

The RNA-Seq data generated in this study have been deposited in the Gene Expression Omnibus (GEO) database under accession code GSE254622 [https://www.ncbi.nlm.nih.gov/geo/query/acc.cgi?acc=GSE254622]. The raw metabolomics data in this study were generated at the Metabolomics Innovation Resource Facility and have been deposited in the Mendeley Data database under accession code DOI: 10.17632/ddxckj7s5b.1 [https://data.mendeley.com/datasets/ddxckj7s5b/1].

Analysis of transcriptomic data from publicly available datasets: Kaplan-Meier curves depicting overall survival (OS) according to CPT1A mRNA expression in breast cancer and HER2+ breast cancer patients were generated from the Gene Expression Profiling Interactive Analysis database (GEPIA2) web-based tool (http://gepia2.cancer-pku.cn/) 97. To assess the prognostic value of CPT1A, patient samples were stratified into two groups, high and low, based on either median or quartile cut-offs. The two patient cohorts were compared using Kaplan-Meier survival plots, and the hazard ratios, calculated using the Cox proportional-hazard model with 95% confidence intervals (CIs), and log-rank p values were displayed on the figure. p-values less than 0.05 were considered significant. Receiver Operating Characteristic (ROC) plots were used to determine whether CPT1A gene expression was implicated as a biomarker for sensitivity or resistance to Trastuzumab in breast cancer. These ROC plots were generated from ROC plotter (https://rocplot.org) by selecting “ROC Plotter for breast cancer” and entering CPT1A as the gene symbol.98 ROC plots were created for both complete pathological response (cPR) and relapse-free survival (RFS) to Anti-HER2 therapy, Trastuzumab.

All findings from this study are available within the article, supplementary information, and source data files or from the corresponding author (W.J.M) upon request. Source data are provided with this paper.

Research involving human participants, their data, or biological material

Policy information about studies with human participants or human data. See also policy information about sex, gender (identity/presentation), and sexual orientation and race, ethnicity and racism.

Reporting on sex and gender	Our research takes into consideration that breast cancer predominately affects women. To reflect the patient demographic of breast cancer, we predominately use female mouse models to accurately depict the epidemiology of the disease, as woman are at a much higher risk of developing breast cancer. However, we also recognize that while rare, 1% of breast cancers are made up of men.
Reporting on race, ethnicity, or other socially relevant groupings	N/A
Population characteristics	N/A
Recruitment	N/A
Ethics oversight	N/A

Note that full information on the approval of the study protocol must also be provided in the manuscript.

Field-specific reporting

Please select the one below that is the best fit for your research. If you are not sure, read the appropriate sections before making your selection.

[x] Life sciences [ ] Behavioural & social sciences [ ] Ecological, evolutionary & environmental sciences

For a reference copy of the document with all sections, see nature.com/documents/nr-reporting-summary-flat.pdf

Life sciences study design

All studies must disclose on these points even when the disclosure is negative.

Sample size	Sample sizes are indicated in each figure, corresponding figure legends, or in the "Statistics and reproducibility" section of the manuscript. For cell culture experiments, the sample size (n) was 3 or 4 biological replicates, per genotype, for animal study experiments n = 6 to 20 mice, per genotype. No power analysis, statistical method, or sample size calculation were used to calculate the sample size, they were determined considering the variations and mean values of the samples, based on our previous experience, or a standard protocol in the field. p-values from statistical tests were used to assess statistical significance and appropriateness of sample sizes. All experiments were reproduced in at least two independent experiments using the indicated biological and technical replicates unless otherwise specified in the figure legends. As we used a stringent definition of response to therapy (tumor onset, weight and growth) in the evaluation of anti-tumor activity of the ketogenic diet (long-chain and medium chain ketogenic diet versus normal chow) and 7.16.4 mAb (versus IgG Control Ab), n= 6 or 10 mice, respectively, was sufficient to reach statistically significant effect as compared to the control group.

Data exclusions	No data was excluded from the analysis and the study.
Replication	To verify the reproducibility of the experimental finding - Multiple samples were obtained per genotype (n = 3 or 4 independent biological replicates, for cell culture and n = 6 to 20 mice, for animal study experiments), each sample was replicated in triplicate or quadruplicate and are depicted on the graph/ analysis. All attempts are replication were successful. In vivo experiments were not replicated for logistic and ethical reasons. Replicates are shown in the figure and outlined in the figure legends.
Randomization	Samples and mice were randomly allocated into control and experimental groups based on the genotype or experimental conditions.<br>For In vivo Ketogenic Diet therapeutic studies and In vivo therapeutic studies (using 7.16.4 mAb and IgG), mice were randomly assigned to treatment groups at the beginning of each experiment.
Blinding	For this study, investigators were blinded to group allocation during data collection and analysis.<br>For in vivo experiments, mice were randomly allocated to treatment groups, and investigators performing drug treatments and tumor measurements were blinded to the group allocation during data collection and data analysis.<br>We used rigorous controls and statistical methods to exclude the potential bias or confounding factors.

Reporting for specific materials, systems and methods

We require information from authors about some types of materials, experimental systems and methods used in many studies. Here, indicate whether each material, system or method listed is relevant to your study. If you are not sure if a list item applies to your research, read the appropriate section before selecting a response.

Materials & experimental systems

n/a	Involved in the study
☐	☒ Antibodies
☐	☒ Eukaryotic cell lines
☒	☐ Palaeontology and archaeology
☐	☒ Animals and other organisms
☒	☐ Clinical data
☒	☐ Dual use research of concern
☒	☐ Plants

Methods

n/a	Involved in the study
☒	☐ ChIP-seq
☐	☒ Flow cytometry
☒	☐ MRI-based neuroimaging

Antibodies

Antibodies used

1. beta-Actin (AC-15), Mouse mAb, Millipore - Cat. No: A5441 (Source No.: 0000167894). Dilution: IB - 1/2000.
2. CD206/ MRC1 (E6T5J), Rabbit mAb, Cell Signaling - Cat. No.:24595, Lot No.: 3. Dilution: IF: 1/400.
3. CD3-epsilon (D4V8L), Rabbit mAb, Cell Signaling - Cat No.: 99940, Lot No.: 1. Dilution: IF: 1/200.
4. CD36, Rabbit anti-CD36-BSA Free pAb, Novus Biologicals - Cat. No.: NB400-144, Lot No.: D105730-2. Dilution: IF: 1/200.
5. CD4 (D7D2Z), Rabbit mAb, Cell Signaling - Cat. No.: 25229, Lot No.: 7. Dilution: IF: 1/200.
6.CD8-alpha (D4W2Z), Cell Signaling - Cat. No.: 98941, Lot No.: 6. Dilution: IF: 1/200.
7. CD31 (PECAM-1) (D8V9E) XP(R), Rabbit mAb, Cell Signaling - Cat No.: 77699, Lot No.: Dilution: IF: 1/200.
8.Cleaved Caspase 3 (D175), Rabbit mAb, Cell Signaling - Cat No.: 9661, Lot No: 47.Dilution: IF: 1/200.
9. CPT1A, Rabbit pAb, Proteintech - Cat. No.: 15184-1-AP, Lot. No.:00061091. Dilution: IF: 1/500, IB: 1/1000.
10. ErbB2, Rabbit pAb, DAKO - Cat. No.: A0485, Lot. No: 41613306. Dilution: IF: 1/200.
11. ErbB2/ c-Neu (Ab3) (CB5), Millipore - Cat. No.: OP15L, Lot. No.: n/a. Dilution: IF: 1/100.
12. F4/80 (D2S3R) XP(R), Rabbit mAb, Cell Signaling - Cat. No.: 70076, Lot. No.: 3. Dilution: IF: 1/200.
13. Glut1 (E4S6I), Rabbit mAb, Cell Signaling - Cat. No.: 73015, Lot No.: 1. Dilution: IF: 1/200, IB: 1000.
14. Hsp60, Mouse mAb, Fisher Scientific - Cat. No.: MA5-15836, Lot. No.: n/a. Dilution: IF: 1/100.
15. KEAP1, Rabbit pAb. Proteintech - Cat. No.: 10503-2-AP, Lot. No. 00118817.Dilution: IB: 1/1000.
16. Ki67 (D3B5), Rabbit mAb, Cell Signaling - Cat. No.: 12202, Lot. No.: 8. Dilution: IF: 1/200.
17. MPC2 (D4I7G), Rabbit mAb, Cell Signaling - Cat. No.: 46141, Lot. No.: 2. Dilution: IB: 1/1000.
18. NK1.1/ CD161 (E6Y9G), Rabbit mAb, Cell Signaling - Cat. No.: 39197, Lot No.:3. Dilution: IF: 1/200.
19. NRF2 (D1Z9C) XP(R), Rabbit mAb, Cell Signaling - Cat. No.:12721l Lot. No.:10. Dilution: IF - 1/200, IB - 1/1000.
20. p-Stat1 (Y701), (D4A7), Rabbit mAb, Cell Signaling - Cat. No.: 9167, Lot. No. 5. Dilution: IF: 1/200.
21. Tomm20 (FL-145), Rabbit pAb, Santa Cruz - Cat. No.: sc-11415, Lot No.: J2815. Dilution: IF: 1/200.
22. alpha-Tubulin (DM1A), Mouse mAb, Cell Signaling - Cat. No.: 3873, Lot No.: 16. Dilution: IB: 1/2000.
23. Vinculin (VIIF9) (7F9), Mouse mAb, Chemicon - Cat. No.: MAB3574, Lot No.: 4015613. Dilution: IB: 1/5000.
24. Alexa Fluor 488 Goat anti-Mouse, Fisher Scientific - Cat. No.: A32723, Lot. No.: n/a. Dilution: IB: 1/1000.
25. Alexa Fluor 555 Goat anti-Rabbit, Fisher Scientific - Cat. No.: A32732, Lot. No.: n/a. Dilution: IB: 1/1000.
26. Alexa Fluor 647 Phalloidin, Fisher Scientific - Cat. No.: A22287, Lot. No.: n/a. Dilution: IB: 1/1000.
27. IRDye 800CW Donkey anti-Rabbit, Li-COR Biosciences - Cat. No.: 925-32213, Lot. No.: D30926-05. Dilution: IB: 1/10000.
28. IRDye 680RD Donkey anti-Mouse, Li-COR Biosciences - Cat. No.: 926-68073, Lot. No.: D30613-05. Dilution: IB: 1/10000.

Where, IF: Immunofluorescence and IB: Immunoblot analysis

Validation

Validations have been performed by the Manufacturer and can be seen on the Manufacturer's website or in the provided technical sheets. Positive and negative cell lines, Activator and Inhibitor Treatment, Tissue Type and Protocol Optimization has been conducted by the manufacturer and our lab to ensure the validity and the application of the following antibodies. (IF: Immunofluorescence and IB: Immunoblot analysis).

1. beta-Actin, IB: 1/2000
Validation statement and information on the manufacturer web site: https://www.sigmaaldrich.com/CA/en/product/sigma/a5441
Monoclonal Anti-β-Actin (mouse IgG1 isotype) is derived from the AC-15 hybridoma produced by the fusion of mouse myeloma cells and splenocytes from an immunized mouse. Actin is one of the most conserved eukaryotic proteins, it is expressed in mammals and birds as at least six isoforms. Four of them represent the differentiation markers of muscle tissues and two are found practically in all cells. There are three α-actins (α-skeletal, α-cardiac, and α-smooth muscle), one β-actin (β-nonmuscle), and two γ-actins (γ-smooth muscle and γ-non-muscle). Actin isoforms show >90% overall sequence homology, but only 50−60% homology in their 18 NH2-terminal residues. The NH2-terminal region of actin appears to be a major antigenic region and may be involved in the interaction of actin with other proteins such as myosin. The antibody can be used for staining of acetone-fixed frozen sections, EM preparations, and microinjection experiments. B5, ethanol, methacam, or Bouin's solutions can be used as fixatives. The epitope recognized by the antibody is resistant to formalin-fixed and paraffin-embedding.Monoclonal Anti β-Actin antibody recognizes an epitope located on the N-terminal end of the β-isoform of actin. The antibody specifically labels β-actin in a wide variety of tissues and species using immunoblotting (42 kDa), immunofluorescent staining of cultured cell lines, and immunohistochemistry. Species Reactivity: heep, carp, feline, chicken, rat, mouse, Hirudo medicinalis, rabbit, canine, pig, human, bovine, guinea pig.

2. CD206/ MRC1, IF: 1/400.
Validation statement and information on the manufacturer web site: https://www.cellsignal.com/products/primary-antibodies/cd206-mrc1-e6t5j-xp-rabbit-mab/24595
Monoclonal antibody is produced by immunizing animals with a synthetic peptide corresponding to residues near the carboxy terminus of mouse CD206/MRC1 protein.CD206/MRC1 (E6T5J) XP® Rabbit mAb recognizes endogenous levels of total CD206/MRC1 protein. This antibody recognizes mouse CD206/MRC1 protein and is also reactive with human CD206/MRC1; however, this antibody is not suggested for immunohistochemical analysis of human tissues. Instead, CD206/MRC1 (E2L9N) Rabbit mAb #91992 is recommended for IHC analysis of human tissue samples. Species Reactivity:Human, Mouse, Rat, Monkey.

3. CD3-epsilon, IF: 1/200.
Validation statement and information on the manufacturer web site: https://www.cellsignal.com/products/primary-antibodies/cd3e-e4t1b-xp-rabbit-mab/78588
Monoclonal antibody is produced by immunizing animals with a synthetic peptide corresponding to residues surrounding Val31 of mouse CD3ε protein.CD3ε (D4V8L) Rabbit mAb recognizes endogenous levels of total mouse CD3ε protein. Non-specific staining in mouse pancreas has been observed. CD3ε (D4V8L) Rabbit mAb may react weakly with human CD3ε, but is not suggested for use in immunohistochemical analysis of human tissues. Instead, CD3ε (D7A6E™) XP® Rabbit mAb #85061 is recommended for IHC analysis of human tissue samples. Species Reactivity: Mouse

4. CD36, IF: 1/200.
Validation statement and information on the manufacturer web site: https://www.novusbio.com/products/cd36-antibody_nb400-144
Rabbit polyclonal. This CD36 Antibody was developed against a synthetic peptide mapping to a region of human CD36 between residues 100-200 [Uniprot# P16671].

5. CD4, IF: 1/200.
Validation statement and information on the manufacturer web site: https://www.cellsignal.com/products/primary-antibodies/cd4-d7d2z-rabbit-mab/25229
Monoclonal antibody is produced by immunizing animals with a synthetic peptide corresponding to residues surrounding Ala232 of mouse CD4 protein.CD4 (D7D2Z) Rabbit mAb recognizes endogenous levels of total mouse and rat CD4 protein. Non-specific staining in mouse kidney and liver has been observed. Species Reactivity: Mouse, Rat, Hamster

6.CD8-alpha, IF: 1/200.
Validation statement and information on the manufacturer web site: https://www.cellsignal.com/products/primary-antibodies/cd8a-d4w2z-xp-174-rabbit-mab/98941
Monoclonal antibody is produced by immunizing animals with a synthetic peptide corresponding to residues surrounding Asp42 of mouse CD8α protein.CD8α (D4W2Z) XP® Rabbit mAb recognizes endogenous levels of total CD8α protein. Species Reactivity: Mouse

7. CD31 (PECAM-1), IF: 1/200.
Validation statement and information on the manufacturer web site: https://www.cellsignal.com/products/primary-antibodies/cd31-pecam-1-d8v9e-xp-rabbit-mab/77699
Monoclonal antibody is produced by immunizing animals with a synthetic peptide corresponding to residues surrounding Ala451 of mouse CD31 (PECAM-1) protein.CD31 (PECAM-1) (D8V9E) XP® Rabbit mAb recognizes endogenous levels of total CD31 (PECAM-1) protein. Species Reactivity: Mouse

8. Cleaved Caspase 3, IF: 1/200.
Validation statement and information on the manufacturer web site: https://www.cellsignal.com/products/primary-antibodies/cleaved-caspase-3-asp175-antibody/9661
Polyclonal antibodies are produced by immunizing animals with a synthetic peptide corresponding to amino-terminal residues adjacent to (Asp175) in human caspase-3.Cleaved Caspase-3 (Asp175) Antibody detects endogenous levels of the large fragment (17/19 kDa) of activated caspase-3 resulting from cleavage adjacent to Asp175. This antibody does not recognize full length caspase-3 or other cleaved caspases. This antibody detects non-specific caspase substrates by western blot. Non-specific labeling may be observed by immunofluorescence in specific sub-types of healthy cells in fixed-frozen tissues (e.g. pancreatic alpha-cells).

Nuclear background may be observed in rat and monkey samples. Species Reactivity: Human, Mouse, Rat, Monkey

9. CPT1A, IF: 1/500 and IB: 1/1000.
Validation statement and information on the manufacturer web site: https://www.ptglab.com/products/CPT1A-Antibody-15184-1-AP.htm
Rabbit Polyclonal. 15184-1-AP targets CPT1A in WB, IP, IHC, IF, FC, CoIP, ELISA applications and shows reactivity with human, mouse, rat samples.

10. ErbB2, IF: 1/200.
Polyclonal Rabbit Anti-Human c-erbB-2 Oncoprotein is intended for use in immunohistochemistry. The antibody labels normal epithelial cells, which generally express c-erbB-2 protein at a very low level. It is a useful tool for the identification of overexpression of c-erbB-2 oncoprotein in a variety of epithelial neoplasms, for example subsets of breast carcinomas, pulmonary adenocarcinomas, colorectal adenocarcinomas, pulmonary squamous and gastric adenocarcinomas, transitional cell carcinomas of the urinary bladder, and endometrial adenocarcinomas. The clinical interpretation of any staining or its absence should be complemented by morphological studies using proper controls and should be evaluated within the context of the patient's clinical history and other diagnostic tests by a qualified pathologisThe antibody labels an intracellular domain of c-erbB2 oncoprotein.

11. ErbB2/ c-Neu (Ab3), IF: 1/100 and IB: 1/1000.
Validation statement and information on the manufacturer web site: https://www.emdmillipore.com/CA/en/product/Anti-c-ErbB2-c-Neu-Ab-3-Mouse-mAb-3B5,EMD_BIO-OP15L?ReferrerURL=https%3A%2F%2Fwww.google.com%2F
Mouse monoclonal. a synthetic peptide (TAENPEYLGLDVPV) corresponding to amino acids 1242-1255 from the C-terminal domain of human c-ErbB2/c-Neu.

12. F4/80, IF: 1/200.
Validation statement and information on the manufacturer web site: https://www.cellsignal.com/products/primary-antibodies/f4-80-d2s9r-xp-rabbit-mab/70076
Monoclonal antibody is produced by immunizing animals with recombinant mouse F4/80 protein.F4/80 (D2S9R) XP® Rabbit mAb recognizes endogenous levels of total F4/80 protein. Species Reactivity: Mouse

13. Glut1, IF: 1/200 and IB: 1/1000.
Validation statement and information on the manufacturer web site: https://www.cellsignal.com/products/primary-antibodies/glut1-e4s6i-rabbit-mab/73015
Monoclonal antibody is produced by immunizing animals with a synthetic peptide corresponding to residues near the carboxy terminus of human Glut1 protein.Glut1 (E4S6I) Rabbit mAb recognizes endogenous levels of total Glut1 protein. This antibody does not cross-react with Glut2, Glut3, or Glut4. Species Reactivity: Human, Mouse, Rat, Monkey.

14. Hsp60, IF: 1/100.
Validation statement and information on the manufacturer web site: https://www.thermofisher.com/antibody/product/HSP60-Antibody-clone-3G8-Monoclonal/MA5-15836
Mouse monoclonal. MA5-15836 targets HSP60 in indirect ELISA, FACS, IF, IHC, and WB applications and shows reactivity with Human, mouse, Non-human primate, and Rat samples. The MA5-15836 immunogen is purified recombinant fragment of human HSP60 expressed in E. Coli. MA5-15836 detects HSP60 which has a predicted molecular weight of approximately 61kDa. Species reactivity: Human, Mouse, Non-human primate, Rat.

15. KEAP1, IB: 1/1000.
Validation statement and information on the manufacturer web site: https://www.ptglab.com/products/KEAP1-Antibody-10503-2-AP.htm
Rabbit polyclonal. 10503-2-AP targets KEAP1 in WB, IP, IHC, IF, CoIP, ELISA applications and shows reactivity with human, mouse samples.

16. Ki67, IF: 1/200.
Validation statement and information on the manufacturer web site: https://www.cellsignal.com/products/primary-antibodies/ki-67-d3b5-rabbit-mab-mouse-preferred-ihc-formulated/12202
Monoclonal antibody is produced by immunizing animals with a recombinant protein specific to the amino terminus of Ki-67 protein.Ki-67 (D3B5) Rabbit mAb (IHC Formulated) recognizes endogenous levels of murine Ki-67 protein. It will also detect endogenous levels of human Ki-67 protein; however, Ki-67 (D2H10) Rabbit mAb #9027 is recommended for the detection of human Ki-67 protein in paraffin-embedded tissues. Species Reactivity: Mouse.

17. MPC2, IB: 1/1000.
Validation statement and information on the manufacturer web site: https://www.cellsignal.com/products/primary-antibodies/mpc2-d4i7g-rabbit-mab/46141
Monoclonal antibody is produced by immunizing animals with a synthetic peptide corresponding to residues surrounding Asn33 of human MPC2 protein. MPC2 (D4I7G) Rabbit mAb recognizes endogenous levels of total MPC2 protein. This antibody does not cross-react with MPC1 protein. Species Reactivity: Human, Mouse, Rat, Monkey.

18. NK1.1/ CD161, IF: 1/200.
Validation statement and information on the manufacturer web site: https://www.cellsignal.com/products/primary-antibodies/nk1-1-cd161-e6y9g-rabbit-mab/39197
Monoclonal antibody is produced by immunizing animals with a synthetic peptide corresponding to residues near the carboxy terminus of mouse NK1.1/CD161 protein.NK1.1/CD161 (E6Y9G) Rabbit mAb recognizes endogenous levels of total CD161c protein from C57BL/6, SJL, and NZW mice, but not from BALB/c and 129S6 mice. Mice from other strains have not been tested. This antibody is predicted to cross-react with CD161b and may cross-react with CD161a protein in some mouse strains. Non-specific staining was

observed in mouse testis by immunohistochemistry. Species Reactivity: Mouse.

19. NRF2, IF: 1/200 and IB: 1/1000.
Validation statement and information on the manufacturer web site: https://www.cellsignal.com/products/primary-antibodies/nrf2-d1z9c-xp-rabbit-mab/12721
Monoclonal antibody is produced by immunizing animals with a synthetic peptide corresponding to residues surrounding Ala275 of human NRF2 protein.NRF2 (D1Z9C) XP® Rabbit mAb recognizes endogenous levels of total NRF2 protein. Species Reactivity: Human, Mouse, Monkey

20. p-Stat1 (Y701), IF: 1/200.
Validation statement and information on the manufacturer web site: https://www.cellsignal.com/products/primary-antibodies/phospho-stat1-tyr701-58d6-rabbit-mab/9167
Monoclonal antibody is produced by immunizing animals with a synthetic phosphopeptide corresponding to residues surrounding Tyr701 of human Stat1.Phospho-Stat1 (Tyr701) (58D6) Rabbit mAb detects endogenous levels of Stat1 only when phosphorylated at tyrosine 701. The antibody detects phosphorylated tyrosine 701 of p91 Stat1 and also the p84 splice variant. It does not cross-react with the corresponding phospho-tyrosines of other Stat proteins. Species Reactivity: Human, Mouse

21. Tomm20, IF: 1/200.
Validation statement and information on the manufacturer web site: https://www.scbt.com/p/tom20-antibody-fl-145
Tom20 Antibody (FL-145) is a rabbit polyclonal IgG; 200 µg/ml.

22. alpha-Tubulin, IB: 1/2000.
Validation statement and information on the manufacturer web site: https://www.cellsignal.com/products/primary-antibodies/a-tubulin-dm1a-mouse-mab/3873
Polyclonal antibodies are produced by immunizing animals with a synthetic peptide corresponding to the sequence of human α-tubulin. Antibodies are purified by protein A and peptide affinity chromatography.The α-Tubulin Antibody detects endogenous levels of total α-tubulin protein, and does not cross-react with recombinant β-tubulin. Species Reactivity: Human, Mouse, Rat, Monkey, D. melanogaster, Bovine

23. Vinculin, IB: 1/5000.
Validation statement and information on the manufacturer web site: https://www.emdmillipore.com/CA/en/product/Anti-Vinculin-Antibody-clone-VIIF9-7F9,MM_NF-MAB3574?ReferrerURL=https%3A%2F%2Fwww.google.com%2F
Mouse Monoclonal. Anti-Vinculin Antibody, clone VIIF9 (7F9) is an antibody against Vinculin for use in IP, WB, IC, IH(P). Species reactivity: Mouse, human, pig, rabbit, monkey, bovine

24. Alexa Fluor 488 Goat anti-Mouse, IF: 1/1000.
Validation statement and information on the manufacturer web site: https://www.thermofisher.com/antibody/product/Goat-anti-Mouse-IgG-H-L-Highly-Cross-Adsorbed-Secondary-Antibody-Polyclonal/A32723
Goat polyclonal. Anti-Mouse secondary antibodies are affinity-purified antibodies with well-characterized specificity for mouse immunoglobulins and are useful in the detection, sorting or purification of its specified target. Secondary antibodies offer increased versatility enabling users to use many detection systems (e.g. HRP, AP, fluorescence). They can also provide greater sensitivity through signal amplification as multiple secondary antibodies can bind to a single primary antibody. Most commonly, secondary antibodies are generated by immunizing the host animal with a pooled population of immunoglobulins from the target species and can be further purified and modified (i.e. immunoaffinity chromatography, antibody fragmentation, label conjugation, etc.) to generate highly specific reagents. Species reactivity: Mouse.

25. Alexa Fluor 555 Goat anti-Rabbit, IF: 1/1000.
Validation statement and information on the manufacturer web site: https://www.thermofisher.com/antibody/product/Goat-anti-Rabbit-IgG-H-L-Highly-Cross-Adsorbed-Secondary-Antibody-Polyclonal/A32732
Goat polyclonal. Anti-Rabbit secondary antibodies are affinity-purified antibodies with well-characterized specificity for rabbit immunoglobulins and are useful in the detection, sorting or purification of its specified target. Secondary antibodies offer increased versatility enabling users to use many detection systems (e.g. HRP, AP, fluorescence). They can also provide greater sensitivity through signal amplification as multiple secondary antibodies can bind to a single primary antibody. Most commonly, secondary antibodies are generated by immunizing the host animal with a pooled population of immunoglobulins from the target species and can be further purified and modified (i.e. immunoaffinity chromatography, antibody fragmentation, label conjugation, etc.) to generate highly specific reagents. Species reactivity: Rabbit.

26. Alexa Fluor 647 Phalloidin, IF: 1/1000.
Validation statement and information on the manufacturer web site: https://www.thermofisher.com/order/catalog/product/A22287
Alexa Fluor 647 phalloidin is a high-affinity F-actin probe conjugated to our bright, photostable, far-red fluorescent Alexa Fluor 647 dye.

27. IRDye 800CW Donkey anti-Rabbit, IB: 1/10000.
Validation statement and information on the manufacturer web site: https://www.licor.com/bio/reagents/irdye-800cw-donkey-anti-rabbit-igg-secondary-antibody
Rabbit IgG. The antibody was isolated by affinity chromatography using antigens coupled to agarose beads. Based on ELISA, this antibody reacts with the heavy and light chains of rabbit IgG, and with the light chains common to most rabbit immunoglobulins. This antibody was tested by ELISA and/or solid-phase adsorbed to ensure minimal cross-reactivity with bovine, chicken, goat, guinea pig, hamster, horse, human, mouse, rat, and sheep serum proteins, but may cross-react with immunoglobulins from other species. The conjugate has been specifically tested and qualified for Western blot and In-Cell Western™ Assay applications.

28. IRDye 680RD Donkey anti-Mouse, IB: 1/10000.

Validation statement and information on the manufacturer web site: https://www.licor.com/bio/reagents/irdye-680rd-donkey-anti-mouse-igg-secondary-antibody
Mouse IgG. The antibody was isolated by affinity chromatography using antigens coupled to agarose beads. Based on immunoelectrophoresis, this antibody reacts with the heavy chains of mouse IgG, and with the light chains common to most mouse immunoglobulins. No reactivity was detected against non-immunoglobulin serum proteins. This antibody was tested by ELISA and/or solid-phase adsorbed to ensure minimal cross-reactivity with bovine, chicken, goat, guinea pig, Syrian hamster, horse, human, rabbit, and sheep serum proteins, but may cross-react with immunoglobulins from other species. The conjugate has been specifically tested and qualified for Western blot and In-Cell Western™ Assay applications.

All antibodies were tested on the mouse (species) samples.

Eukaryotic cell lines

Policy information about cell lines and Sex and Gender in Research

Cell line source(s)	Mammary tumors at 8 weeks post-palpation were excised from female MMTV-NIC and MMTV-NIC/ CPT1A flx/flx mice, dissociated in collagenase B (Roche, 11088831001)/Dispase II (Roche, 4942078001) (2.4 mg/ml each) for 2h at 37 degrees C, washed three times with PBS/1mM EDTA and plated in Complete Media, consisting of DMEM (Wisent, 319-005-CL) supplemented with 2% FBS (Wisent, 080-150), 5ng/ml EGF (Winsent, 511-110-UM) 1 µg/ml Hydrocortisone (Sigma, H4001), 5 µg/ml Insulin (Wisent, 511-016-UG), 35 µg/ml Bovine Pituitary Extract (BPE – Hammond CellTech, 1078-NZ) and 50 ug/ml Penicillin/Streptomycin (Wisent, 450-200-EL). Cells were maintained in a humidified, 5% CO2, 37 degrees C incubator in Complete Media. The human cell lines 293T (CRL-3216) was purchased from ATCC, used at early passage, and maintained in DMEM with 10% FBS.
Authentication	Primary mammary epithelial tumor-derived cell lines were validated by PCR-based genotyping (oligonucleotide details in Supplementary Table S1) analysis, to ensure that all cell lines contained the CPT1A flx/flx, Neu and Cre alleles and by immunoblotting to detect Cpt1a expression. For knockdown studies (NRF2, KEAP1 and MPC2), efficient ablation of the protein was determined by immunoblot analysis. 293T were from ATCC directly, and were not authenticated by our lab.
Mycoplasma contamination	All cells used in this study were negative for mycoplasma contamination. All cell lines were tested biweekly for mycoplasma using the MycoAlert Kit (Lonza, LT07-118). Once thawed were kept in culture for a maximum of 10 passages
Commonly misidentified lines (See ICLAC register)	No commonly misidentified lines were used.

Animals and other research organisms

Policy information about studies involving animals; ARRIVE guidelines recommended for reporting animal research, and Sex and Gender in Research

Laboratory animals	MMTV-NIC and Cpt1a conditional mice were bred on a pure FVB/N background.<br><br>Mouse Housing conditions: All transgenic mice were generated through an in-house breeding program. Female littermates were group housed in autoclaved cages under specific pathogen-free conditions with ad libitum access to food and water, as well as appropriate and sufficient nesting and bedding material. Mice had a 12 hour cycle of light and darkness. Mouse rooms and cages were well-ventilated and kept at a temperature of 20-24 degrees Celsius, with a relative humidity of 45-65%.<br><br>For this study, we used the following mouse strains (Species - Mus musculus):<br>- Transgenic models: Cohorts of female MMTV-NIC mice (Genetic background - FVB/Ncrl, Strain code: 207, Charles River) carrying wild-type or conditional alleles of Cpt1a (n = 20 per genotype) were monitored for mammary tumor formation by twice weekly palpation. Tumor monitoring began on 8-week-old mice (2 months) and continued until the end of the experiments at 10 months. Once detected, tumors were measured weekly using calipers until they had reached a volume of 2.5 cm3 in size for a single mass or a total volume of 5 cm3 for multifocal tumors, at which point mice were euthanized in accordance with approved facility protocols.<br><br>- In vivo Ketogenic Diet Therapeutic Study: 1 x 10^6 NIC/Cpt1a+/+ and NIC/Cpt1aL/L cells were suspended in 30 µl of PBS and injected into the mammary fat pads of 12-week-old female FVB/N mice (Genetic background - FVB/Ncrl, Strain code: 207, Charles River). Mice were kept on a standard rodent diet (Teklad, Inotiv, 2920X), Long-chain Ketogenic diet (BioServ, F3666) and Medium-chain Ketogenic diet (Bioserv, F10595). Tumor monitoring began on 8-week-old mice (2 months) and continued until the end of the experiment 9 months.<br><br>- In vivo therapeutic studies: 1 x 10^6 NIC/Cpt1a+/+ and NIC/Cpt1aL/L cells were suspended in 30 µl of PBS and injected into the mammary fat pads of 8-week-old female FVB/N mice (Genetic background - FVB/Ncrl, Strain code: 207, Charles River). Female mice were randomly assigned to treatment groups, IgG or 7.16.4 mAb (n = 10 mice per treatment group) and monitored for tumor growth by twice-weekly palpation. Tumor monitoring began on 8-week-old mice (2 months) and continued until the end of the experiment 5 months.
Wild animals	The study did not involve wild animals.
Reporting on sex	Breast Cancer is a disease that predominately afflicts female patients. As such, animal studies (Transgenic models, Orthotopic allografts, In vivo Ketogenic Diet therapeutic studies, In vivo therapeutic studies) were only conducted on female FVB/N mice. All primary mammary epithelial tumor-derived cell lins models used in this study were of female origin. No information/data about gender were collected.

Field-collected samples	The study did not involve samples collected from the field.
Ethics oversight	Experiments involving mice were conducted in accordance with McGill University and Canadian Council on Animal Care (CCAC) ethical guidelines under a protocol (MCGL-5518) approved by the McGill University Downtown Campus Facility Animal Care Committee (FACC), a branch of the McGill University Animal Care Committee (UACC), Montreal, QC, Canada.

Note that full information on the approval of the study protocol must also be provided in the manuscript.

Plants

Seed stocks	N/A
Novel plant genotypes	N/A
Authentication	N/A

Flow Cytometry

Plots

Confirm that:

- [x] The axis labels state the marker and fluorochrome used (e.g. CD4-FITC).
- [x] The axis scales are clearly visible. Include numbers along axes only for bottom left plot of group (a 'group' is an analysis of identical markers).
- [x] All plots are contour plots with outliers or pseudocolor plots.
- [x] A numerical value for number of cells or percentage (with statistics) is provided.

Methodology

Sample preparation	Cells were treated with 2M of cell membrane-permeant fluorophore, BODIPY 493/503 (4,4-Difluoro-1,3,5,7,8-Pentamethyl-4-Bora-3a,4a-Diaza-s¬-Indacene) (Thermo Fischer Scientific, #D3922) or BODIPY FL C16 (4,4-Difluoro-5,7-Dimethyl-4-Bora-3a,4a-Diaza-s-Indacene-3-Hexadecanoic Acid) (Thermo Fischer Scientific, #D3821) for 30 minutes at 37 C to stain for lipid droplets. Samples were washed with PBS to remove staining solution, trypsinized to generate single cell suspension and centrifuged at 800 x g for 3 minutes. Subsequently, cells were washed with FACS buffer (1X PBS with 2 mM EDTA and 2% FBS), assed through a 70m strainer and stained with viability dye eFluorYM 506 (eBioscience, 65-00866-18) for 10 minutes on ice.
Instrument	BD LSR Fortessa 5L (BD Bioscience, H649225B4076)<br>5 Lasers (355; 405; 488; 561; 640); 18 detectors (3UV; 6V; 2B; 4YG; 3R) + FSC; SSC-B
Software	FACSDiva Software (Version 8) (BD Biosciences) for data collection and FlowJo Software (Ashland, OR, USA) for data analysis.
Cell population abundance	A minimum of 100 000 events per sample was acquired using a LSR Fortessa Flow Cytometer with FACSDiva Software (Version 8) (BD Biosciences) in slow rate mode to avoid doublets. Cell debris and aggregates were excluded from the analysis using pulse processing SSC-H vs SSC-W
Gating strategy	The first gating strategy, forward- and side-Scatters (FSC and SSC), i.e., representing the cell size and granularity respectively. Then, FSC-A/FSC-H to exclude events that could represent more than one cell. We used viability dye fluor 506/FSC-A to exclude live/dead gate cells were negative efluor 506 cells represent the fraction of viable cells. We then generated a histogram representing Alexa Fluor 488 positive cells for either BODIPY 493/503 or BODIPY FL C16.

- [x] Tick this box to confirm that a figure exemplifying the gating strategy is provided in the Supplementary Information.
